# Giant Optical Anisotropy in a Natural van der Waals Hyperbolic Crystal for Visible Light Low-Loss Polarization Control

**DOI:** 10.1021/acsnano.5c07323

**Published:** 2025-07-01

**Authors:** Nicola Melchioni, Andrea Mancini, Lin Nan, Anastasiia Efimova, Giacomo Venturi, Antonio Ambrosio

**Affiliations:** † Centre for Nano Science and Technology, 403543 Fondazione Istituto Italiano di Tecnologia Via Rubattino 81, Milano 20134, Italy; ‡ Physics Department, Politecnico di Milano, Piazza Leonardo da Vinci 32, Milano 20134, Italy

**Keywords:** in-plane hyperbolicity, ultrathin polarization devices, optical anisotropy, polarization control, low loss, layered van der Waals materials

## Abstract

Optically anisotropic bidimensional crystals offer a promising path toward compact, lithography-free polarization control in integrated photonic devices. However, most materials exhibit only modest optical anisotropy, requiring long propagation lengths to effectively modify the polarization state of light, hindering miniaturization and integration. While some materials achieve strong polarization extinction via directional absorption, this often comes at the cost of high optical losses, limiting their practical use. Here, we investigate the van der Waals crystal MoOCl_2_ that exhibits broadband in-plane hyperbolicity spanning the visible to near-infrared spectrum, driven by a Drude-like response. Thin MoOCl_2_ (∼100–200 nm) flakes achieve high reflectivity (>80%) along the metallic axis and strong transmission (>50%) along the orthogonal dielectric axis, enabling polarization extinction with minimal loss. From polarization-resolved transmission and reflection measurements, we extract an in-plane dielectric permittivity anisotropy exceeding |Δ­(ε_∥_)| > 10 for wavelengths above 600 nm, among the highest reported to date. We further demonstrate the integration of a MoOCl_2_ flake directly onto a connected optical fiber to realize a broadband, ultrathin polarizer. These results establish MoOCl_2_ as a compelling platform for low-loss, miniaturized polarization control in next-generation photonic systems.

## Graphical abstract

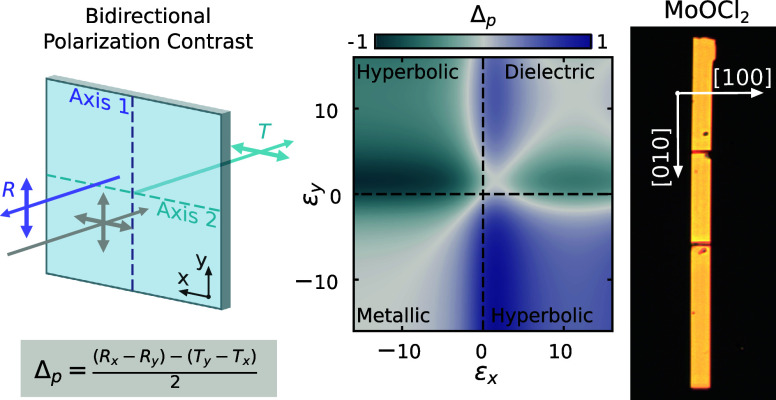

## Introduction

Control over light polarization is fundamental to a range of optical technologies, from information multiplexing in optical fibers^1^ and quantum cryptography based on polarization-entangled photon states^2,3^ to liquid crystal-based displays^4^ and spatial light modulators.^5^ Polarization control relies on anisotropic light–matter interactions that yield distinct reflection or transmission behaviors along orthogonal directions. For example, in wire-grid polarizers—the most broadband polarizing devices currently available—light polarized parallel to the finely spaced metal wires is strongly absorbed, while the orthogonal component interacts weakly and is transmitted.^6^ More recently, metasurfaces^7–9^ and liquid crystal devices^4,10^ have enabled advanced functionalities such as phase modulation^11,12^ and dynamic control.^13^ However, reliance on complex nanofabrication processes and inherent optical losses—particularly in the visible range—has driven the search for anisotropic natural materials as a low-cost alternative for polarization control in integrated devices.^14^

In this context, layered van der Waals (vdW) crystals have attracted significant attention due to their inherent high anisotropy along the out-of-plane direction and their potential for device miniaturization enabled by their two-dimensional structure.^14–19^ However, the thinness of vdW flakes makes it difficult to effectively utilize their out-of-plane optical response. As a result, biaxial crystals exhibiting in-plane optical anisotropy have become more attractive. Several of such materials, including black phosphorus (BP),^20^ ReS_2_,^21^ and As_2_S_3_,^22^ have been investigated. Nonetheless, their moderate anisotropy hinders the complete extinction of one polarization component in either reflection or transmission. Only recently, complete polarization extinction has been achieved in materials with polarization-selective absorption,^19,23^ though their high intrinsic losses present a barrier to practical deployment in photonic systems.

Complete polarization extinction ratio without dissipation can only be achieved when one polarization is perfectly reflected while the orthogonal one is fully transmitted (Figure 1a). This behavior is exemplified by bulky polarizing beam splitters or wire-grid polarizers at low frequencies, where metallic losses are negligible.^24^ As a figure of merit for this ideal condition, we introduce the ″bidirectional polarization contrast″, defined as

1 $$\Delta_{P}=\frac{\left(R_{i}-R_{j}\right)-\left(T_{j}-T_{i}\right)}{2}$$

where *i*, *j* indicate orthogonal in-plane crystal axes. Δ_*P*_ reaches its maximum value only in the lossless limit, when both the reflection (*R*) and transmission (*T*) coefficients approach unity, corresponding to a 100% polarization ratio in each direction.

**Figure 1:**
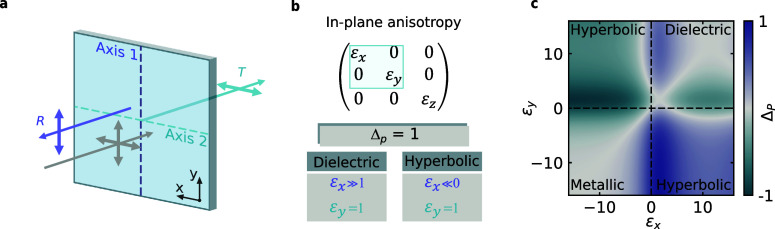
Polarization control with highly anisotropic materials. (a) In an anisotropic material, the polarization of light is manipulated by exploiting the different refractive indexes along the crystal axes. High extinction ratios for both reflected and transmitted light can be achieved simultaneously with low optical losses. (b) At a single air interface, high extinction ratio can be achieved in two low-loss distinct conditions of in-plane anisotropy: highly anisotropic dielectrics with very high refractive index or hyperbolic materials with a strong metallic character. In both cases complete transmission along one axis requires ε = 1 for complete reflection suppression. (c) Plot of Δ_*P*_ at an illumination wavelength λ_0_ for a uniaxial material of thickness λ_0_/10 with dielectric permittivity ε_*x*_ and ε_*y*_ along the corresponding optical axis. Purely real permittivities are used in the plot.

In natural crystals, near-unity reflection can be realized in two distinct ways. One approach is to use a dielectric with a very high refractive index (ε_*x*_ ≫ 1), Figure 1b); however, obtaining 80% reflectivity from a single air interface already demands a refractive index of approximately *n* ∼ 4.5, close to the upper limit of what is found in natural crystals at visible and near-IR frequencies.^25^ Alternatively, one can exploit the strong reflectivity of materials with metallic optical behavior (ε_*x*_ ≪ 0, Figure 1b). However, to achieve at the same time high transmission along the orthogonal axis, a lossless dielectric response is required. In this context, hyperbolic materials that have permittivity elements with opposite signs of the real part (metallic and dielectric along orthogonal axes) offer an ideal platform for achieving high Δ_*P*_.^26^ This advantage is illustrated in Figure 1c, which shows the calculated Δ_*P*_ in the absence of losses for a film with a thickness of λ_0_/10 at varying permittivity values.

Nevertheless, when considering a real hyperbolic material, the origin of its anisotropy crucially influences the spectral range in which hyperbolic dispersion emerges. For example, materials such as MoO_3_ ^27–29^ and CaCO_3_ ^30,31^ exhibit hyperbolic behavior due to phonon resonances, typically confined to the infrared. Materials like NbOCl_2_ display hyperbolicity arising from interband electronic transitions, which generally occur in the blue to ultraviolet spectral region.^23^ Furthermore, these resonance-driven hyperbolic responses tend to occur within narrow spectral bands.^32,33^ A third option is realized in materials exhibiting anisotropic Drude responses due to different plasma frequencies along different crystallographic axes.^34^ Such Drude-driven hyperbolicity can span a broad spectral range, without being inherently restricted to specific regions of the electromagnetic spectrum. Moreover, while resonant mechanisms typically coincide with strong absorption edges, materials exhibiting Drude-like responses are not necessarily linked to pronounced absorption peaks, thereby allowing for lower optical losses in the system.

In this work, we demonstrate that MoOCl_2_, the first van der Waals crystal with a Drude-originated broad hyperbolic region spanning the visible and near-IR,^35^ exhibits strong optical anisotropy in both reflection and transmission while maintaining low optical losses. As a result, even nanometer-scale flakes of MoOCl_2_ achieve a value of Δ_*P*_ ∼ 0.8. We experimentally determine the in-plane dielectric permittivity tensor of MoOCl_2_ and report a record-high difference between its principal components, with |Δ­(ε_∥_)| > 10 for wavelengths above 600 nm in the visible and near-IR ranges. Our findings establish hyperbolic crystals as promising building blocks for miniaturized polarizing optical components, with MoOCl_2_ currently representing the only known hyperbolic van der Waals material operating in the visible range. To illustrate this potential, we theoretically study the use of thin flakes of MoOCl_2_ as nanoscale polarizing beam splitters. Moreover, we experimentally integrate MoOCl_2_ as an ultrathin polarizer on a commercial optical fiber, demonstrating its seamless compatibility with miniaturized and on-chip photonic devices.

## Results

The in-plane crystal structure of MoOCl_2_ features Mo atoms bonded to four Cl atoms and to two O atoms, forming a system of interconnected octahedra (Figure 2a). Peierls distortions are visible in the crystal structure, resulting in a dimerization of Mo atoms. Such dimerization induces orbital-selective Peierls transitions^36^ that significantly alter the electronic bands, creating localized states along the Mo–Cl [010] axis and highly delocalized states along the Mo–O [100] axis. This difference in polarizability of electrons reflects in a strongly anisotropic crystal lattice,^37^ which can be viewed as a system of 1D metallic Mo–O–Mo chains interlinked by Cl atoms.^35^ The resulting quasi-1D crystal structure yields an in-plane hyperbolic dielectric tensor, giving rise to unique properties in MoOCl_2_, such as the propagation of hyperbolic plasmon polaritons^34,38^ and increased magnetoresistance.^39^ As a further consequence of the anisotropic lattice, the crystal exfoliates in regular elongated shapes, with the short and long edges aligned with the [100] and [010] axes, respectively (Figure 2b).

**Figure 2:**
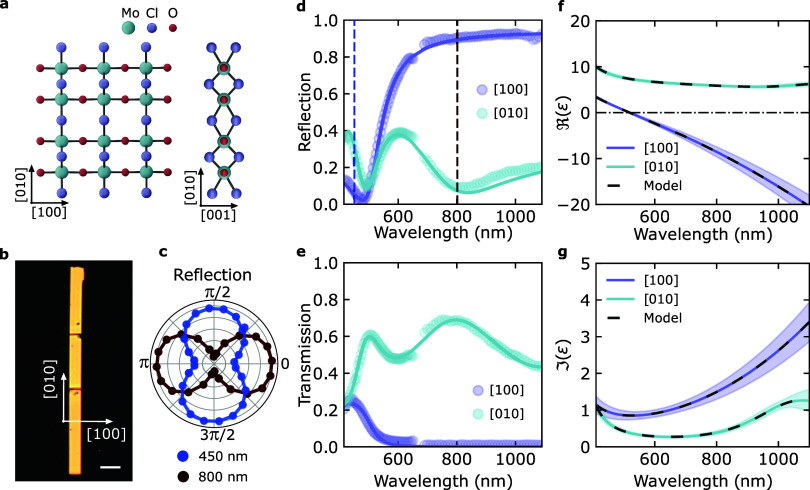
Crystal structure and optical properties of MoOCl_2_. (a) Crystal structure of MoOCl_2_. Crystalline directions are indicated by arrows. (b) Optical image of a MoOCl_2_ flake exfoliated on top of SiO_2_. Corresponding crystal directions are overimposed. Scalebar is 25 μm. (c) Reflection of the flake in (b) as a function of the impinging polarization angle for two different wavelengths. (d,e) Reflectance (d) and transmittance (e) of a 173 nm thick flake with polarization along the metallic (purple dots) and dielectric (light blue dots) optical axes. Solid lines are the fitting of the curves. (f,g) Averaged real part (f) and imaginary part (g) of the dielectric function along the metallic (purple line solid line) and dielectric (light blue solid line) directions extracted from the fits of reflection and transmission. Shaded area represents the standard deviation around the average. The dashed gray line is the fitting of the average value with a Drude-Lorentz model.

To retrieve the dielectric function of MoOCl_2_ with high accuracy, we performed polarization-dependent measurements of both transmission and reflection on flakes of various thicknesses (see Supporting Information S1 and S5). Our experiments unveil a clear anisotropy that also shifts sign with frequency (Figure 2c). Indeed, both the reflection (Figure 2d) and transmission (Figure 2e) spectra taken on a 173 nm thick flake show a strong dependence on the polarization direction. In particular, the [100] axis shows metallic behavior with near-unity reflectivity and almost no transmission for wavelengths above ∼600 nm, while the [010] axis shows low reflectivity and high transmission featuring oscillations compatible with a Fabry–Perot dielectric cavity. The experimental determination of the in-plane dielectric tensor is performed by fitting at the same time both reflection and transmission spectra through a normal-incidence three-layer system model (see Methods). It is important to note that such model is an approximation of the real experimental condition, where light is reflected and transmitted at nonzero angles due to the objective focus. However, the low numerical apertures (NAs) of the used objectives ensure that these effects are negligible, justifying the normal-incidence approximation (see also Supporting Information S3). In the fitting, we fixed the thickness of the flakes as determined from atomic force microscopy (AFM) measurements (see Supporting Information S4). The parameters of the permittivity were extracted by fitting with a Drude-Lorentz model the average ε­(ω) obtained from individual flakes (see Methods, additional spectra are reported in Supporting Information S5).

As visible in Figure 2f–g, the permittivity along the [100] axis is mainly determined by a Drude response with plasma frequency ω_p_ = 5.75 eV. This value is higher than that extracted from other experimental measurements on a bulk crystal,^38^ but still lower than theoretical predictions that neglect electronic correlations^35^ (see Supporting Information S6 for a visual comparison). Conversely, the [010] axis shows dielectric behavior with two Lorentz oscillators at ω_0,1_ = 3.79 eV and ω_0,2_ = 1.16 eV respectively, both associated with the electronic transition between the valence and conduction bands of MoOCl_2_ at different points of the Brillouin zone.^35,40^ The effect of such transitions on the optical response of the [100] axis is limited, but not negligible (the parameters of the whole model are reported in Supporting Information Table S1). The dielectric tensor extracted from our measurements accurately reproduces the optical response of MoOCl_2_ flakes (see Supporting Information S7), thereby confirming its validity. Further confirmation is provided by comparison with experimental dispersion of thin-film hyperbolic plasmons reported in previous works (see Supporting Information S8).

From the transmission and reflection spectra, we computed the anisotropic optical response using |Δ*R*| = |*R*_*x*_ – *R*_*y*_| and |Δ*T*| = |*T*_*x*_ – *T*_*y*_|. In reflection (Figure 3a), |Δ*R*| exceeds 0.5 in most of the vis-NIR region, peaking above >0.7 at around 800 nm. Notably, Δ*R* changes sign at approximately 517 nm, a thickness-dependent crossover point with potential applications in polarized detection and optical switching.^41–43^ In transmission, |Δ*T*| remains around 0.5 in a broadband range between 550 and 1000 nm. The lower value of Δ*T* can be attributed to the simultaneous presence of nonzero reflectivity and absorption along the dielectric axis. A commonly used metric to quantify anisotropy is the normalized polarization contrast, defined as *PC*(*R*) = (*R*_*x*_ – *R*_*y*_)/(*R*_*x*_ + *R*_*y*_) (and equivalently for transmission). While this metric is often referred to as “linear dichroism” in the literature,^19,23^ we avoid this term here to prevent confusion with another common definition: the difference in the imaginary parts of the refractive index, κ_*x*_ – κ_*y*_, also known as linear dichroism.^44^ MoOCl_2_ simultaneously reaches values of *PC*(*R*) ∼ 0.8 and *PC*(*T*) > 0.99 in the visible region, the highest reported to date (see Supporting Information S9).

**Figure 3:**
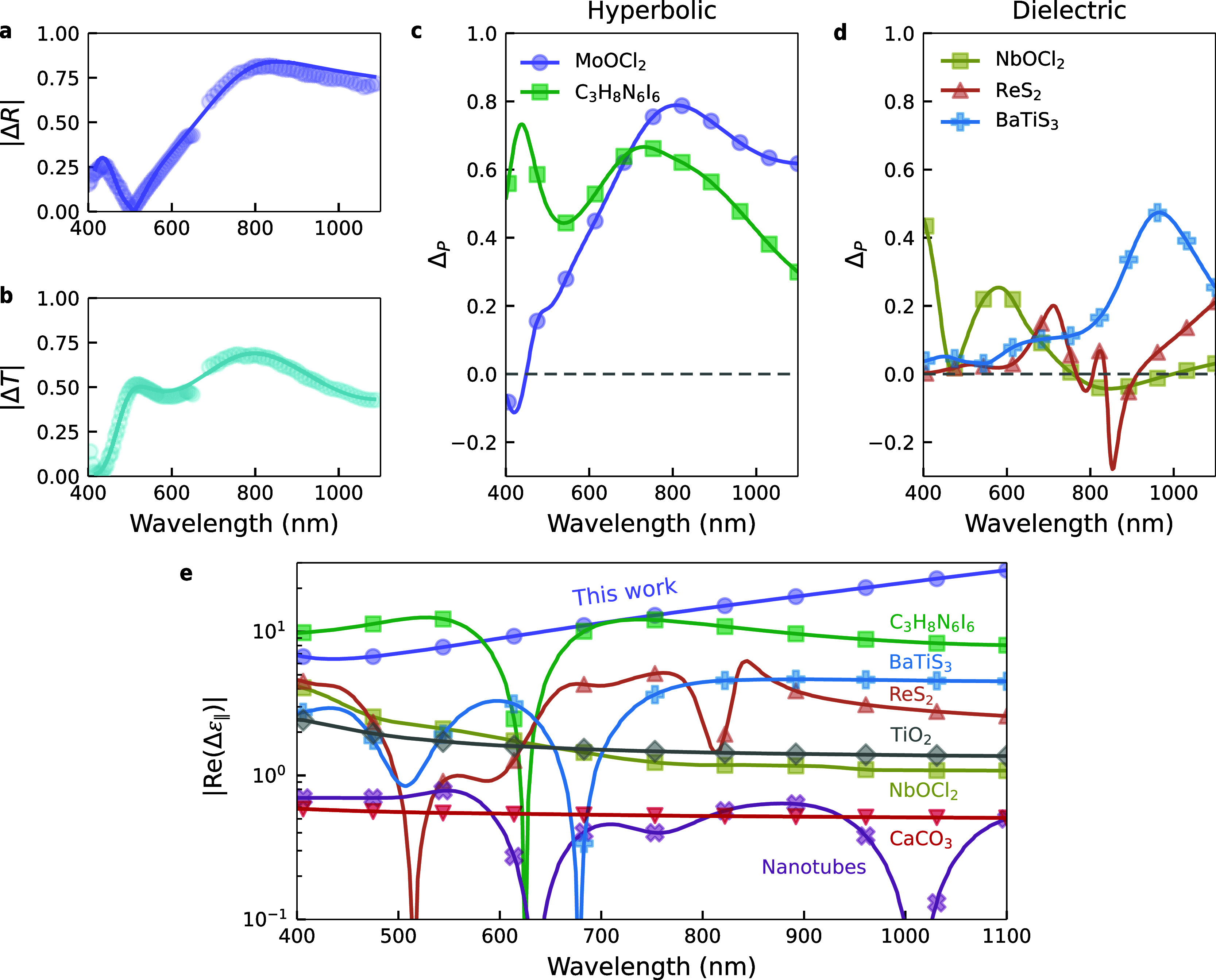
Comparison of the optical anisotropy in MoOCl_2_ and other birefringent materials. (a,b) Absolute value of the difference in reflection (a) and transmission (b) along the crystal axes of MoOCl_2_ measured on the same flake shown in Figure 2d–e. Solid line is a fit to the data. (c,d) Comparison of Δ_*P*_ for a 173 nm thick MoOCl_2_ with other hyperbolic^32^ (c) and dielectric^23,45,46^ (d) materials of equal thickness. e, Comparison of the in-plane permittiviy anisotropy |Re­(Δε_∥_)| of MoOCl_2_ (purple circles) with the materials discussed above, as well as other relevant birefringent materials.^44,47,48^

The experimental Δ_*P*_ of a 173 nm thick MoOCl_2_ exceeds 0.5 for wavelengths longer than 650 nm, reaching a maximum of Δ_*P*_ ∼ 0.8 for 800 nm (Figure 3c). This value surpasses even the one reached in C_3_H_8_N_6_I_6_, a recently studied solution-processable natural bulk crystal that is also hyperbolic.^32^ In contrast, most common anisotropic dielectrics of equal thickness only reach values Δ_*P*_ < 0.5 (Figure 3d), limited by absorption or the lack of considerable polarization extinction in both refection and transmission.^23,44–48^ Although a value of Δ_*P*_ = 1 corresponds to the lossless case, intermediate values cannot clearly discern between the presence of absorption or lower polarization extinction ratio. For a more direct visualization of these competing effects, a plot of |Δ*R*| versus |Δ*T*| can be used (see Supporting Information S10). This representation also confirms the superior performance of hyperbolic materials compared to dielectrics. In addition, an alternative metric to Δ_*P*_ for the evaluation of the overall optical anisotropy is the average of both normalized polarization contrasts (*PC*(*R*) – *PC*(*T*))/2, (see Supporting Information S11). With this figure of merit, hyperbolic materials still outperform standard dielectrics, but the effect of optical losses is masked by the normalization. We therefore chose to use Δ_*P*_ for a more accurate evaluation of materials performance.

Although Δ_*P*_ is a reliable measure of optical anisotropy, it depends on both film thickness and substrate (see Supporting Information S12), making it unsuitable for a comprehensive comparison of different materials. A commonly used metric to quantify the intrinsic optical anisotropy of a material is the birefringence (i.e., the difference in the real part of the complex refractive index), with MoOCl_2_ demonstrating record-high values across a broad range in the visible and near-infrared spectra (see Supporting Information S13). The in-plane birefringence of MoOCl_2_ is higher than observed in all other known materials, whether bulk, as the commercially used rutile^48^ or BaTiS_3_,^46^ or 2D, like the recently investigated NbOCl_2_.^23^ It also surpasses other visible-hyperbolic crystals^32,49^ and semiartificial materials such as aligned carbon nanotubes.^44^ While birefringence is effective for comparing low-loss dielectrics, it falls short in distinguishing between metallic and dielectric responses as two crystal axes can have the same *n* but very different κ, leading to strong optical anisotropy but no birefringence. As this distinction is key in hyperbolic materials, we believe the difference in the real part of the permittivity |Δε_∥_| = |ε_*x*_ – ε_*y*_| to be better suited for a fair material comparison (Figure 3e). Also in the case of this alternative metric, MoOCl_2_ features record-high values at visible and near-IR frequencies, with C_3_H_8_N_6_I_6_ the only material with comparable properties, again highlighting the advantage of hyperbolic crystals in maximizing optical anisotropy.

The high value of Δ_*P*_ in the technologically relevant vis-NIR region of the spectrum establishes MoOCl_2_ as a benchmark 2D material for nanometer-scale broadband polarizing components, offering high polarization efficiency and low power loss. Among other applications, MoOCl_2_ could be employed as a polarizing beam splitter (PBS), whereas standard implementations are either thin narrow-band plates or very bulky beam splitter cubes. To spatially separate the reflected light from the incoming beam, a tilt angle must be introduced, resulting in different optical responses for s and p polarizations (see sketch in Figure 4a). The use of a tilt angle also allows the complete suppression of the p—polarized reflection component along the dielectric [010] direction at the Brewster angle (Figure 4b), as evidenced by the simulated reflectivity dip for a 100 nm MoOCl_2_ film in air at λ̃ = 700 nm, which we calculated using the transfer-matrix method.^50^ At the same time, the transmission along the [100] axis for the complementary s—polarized light is generally low because of the metallic response and is further suppressed for high incidence angles (Figure 4c). Hyperbolicity can then be leveraged to obtain a unique combination of strong polarization selectivity in both reflection and transmission by making use of the Brewster angle along the dielectric axis and the high reflectivity of the metallic direction. As suppression of the reflectivity along the dielectric axis is key to obtain an efficient PBS, it is useful to understand its behavior as a function of film thickness and incidence angle at λ̃. Reflectivity minima can be either obtained for specific thicknesses thanks to destructive Fabry–Perot interference, weakly dependent on the incidence angle, or at the Brewster condition independently of film thickness (Figure 4d). To quantify the performance of a polarizing element it is customary to use the so-called polarization-extinction ratio (ER), which we define here in reflection and transmission as *ER*_R_ = *R*_s_/*R*_p_ and *ER*_T_ = *T*_p_/*T*_s_. The calculated ER is plotted in Figure 4e for a 160 nm film, being this the thickness at which the minimum of the p-polarized reflectivity is found in Figure 4d. In Figure 4e, we report the values of ER in reflection (blue curves) and transmission (purple curves) at two different incident angles, θ_inc_ = 45° (dashed lines) and at the Brewster angle θ_inc_ = 68.5° (solid lines). The advantage of using the Brewster condition is evidenced by the stronger reflection ER. Due to dispersion, the Brewster angle changes with wavelength, leading to a peak at the design wavelength λ = 700 nm in Figure 4e. Importantly, for the specific thickness chosen, the ER in both reflection and transmission is over 10^3^ in a broad wavelength range 580–830 nm. For comparison, commercial bulky polarizing beam splitters made through dielectric coatings, achieve an *ER* > 10^3^ only in transmission, while in reflection they only reach an *ER* < 10^2^. Tuning the reflectivity ER peak requires matching the Brewster angle as it shifts due to dispersion, which is not ideal for alignment purposes when changing optical element. Instead, one can try to operate at a fixed angle and compensate the increased p reflectivity by changing the film thickness in order to achieve destructive interference with the reflection at the bottom interface. This process is shown in Figure 4d, where we fix θ_inc_ = 68.5° and tune the film thickness to achieve a maximum in the reflection ER. In this way, we show that simultaneous reflection and transmission values of *ER* > 10^3^ can be achieved over the whole 500–1100 nm range for films thinner than 250 nm. Overall, we demonstrated that by leveraging the unique properties of hyperbolic materials it is possible to obtain ultrathin PBSs with simultaneously high values of polarization extinction ratios in reflection and transmission.

**Figure 4:**
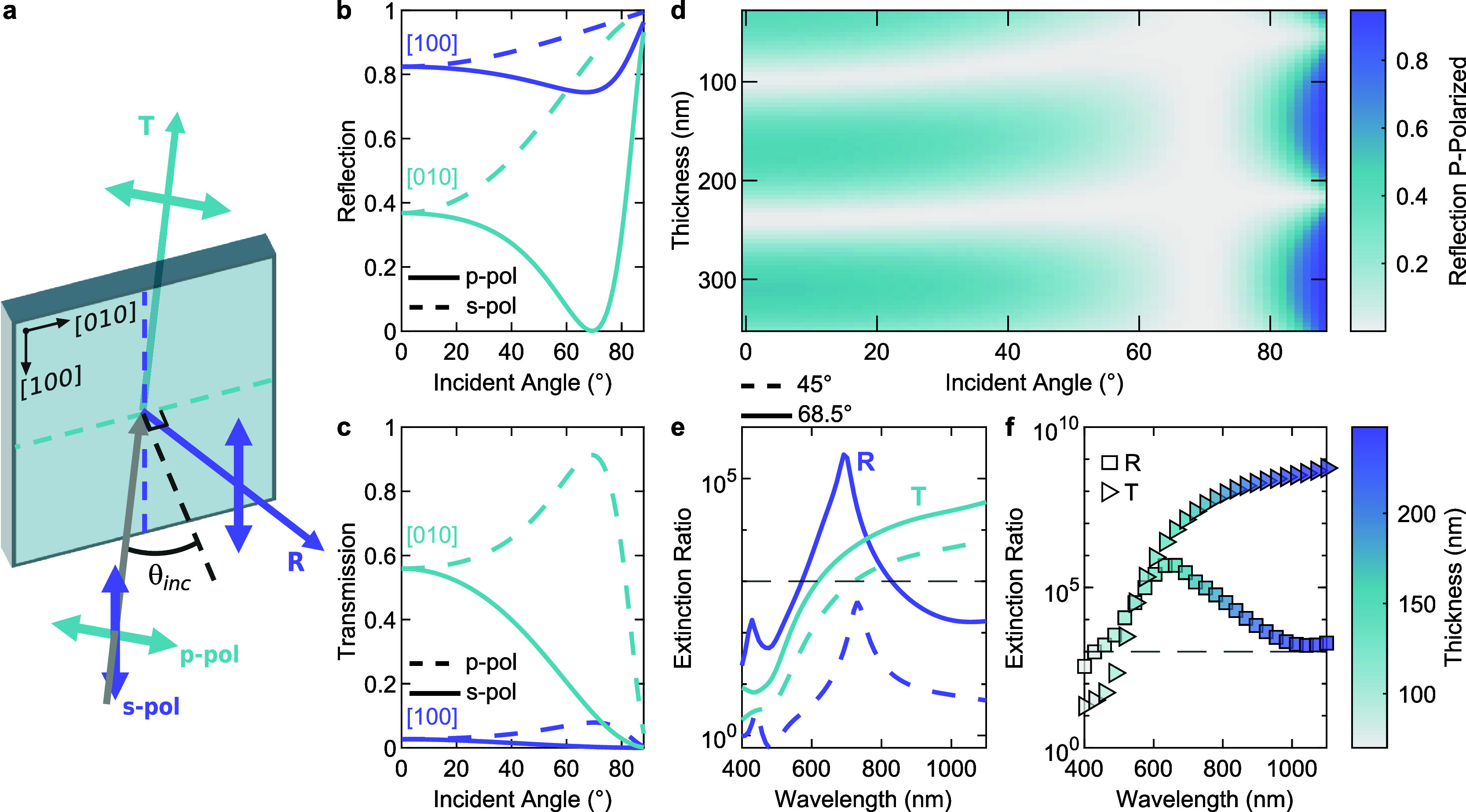
MoOCl_2_ as an ultrathin polarizing beam splitter. (a) A thin flake of MoOCl_2_ can be used as a polarizing beam splitter. The optimal configuration is achieved when p-polarized light is aligned along the [010] axis, allowing the use of Brewster’s angle to suppress reflectivity along that direction. (b,c) Reflectivity (b) and transmission (c) of a 100 nm thick MoOCl_2_ flake for all the possible alignement of p-polarized (solid lines) and s-polarized (dashed lines) light with respect to the crystal axes (indicated by the colors) at a fixed wavelength of λ̃ = 700 nm. (d) Reflectivity of p-polarized light along the [010] axis of the crystal at λ̃. e Extinction ratio in reflection (purple) and transmission (light blue) for light impinging on a 160 nm thick crystal at the Brewster’s angle (solid lines) or at 45° (dashed lines). The gray horizontal dashed line indicates *ER* = 10^3^. (f) The thickness of the flake (in the colorbar) can be chosen to simultaneously maximize the *ER* in reflection (squares) and transmission (triangles) at specific wavelengths and fixed θ_inc_, allowing the design of specific polarizing beam splitters across the whole visible spectrum. The gray dashed line indicates *ER* = 10^3^.

Finally, as a practical example of the ready integrability of MoOCl_2_ in a functional miniaturized optical system, we fabricated a broadband polarizing fiber by transferring exfoliated flakes onto the ferrule end of an optical fiber (Figure 5a). We use a commercially available single-mode (SM) optical fiber with a cladding diameter of 125 μm and a mode diameter between 3.3 and 4.6 μm, depending on the wavelength (Figure 5b). A MoOCl_2_ flake is transferred onto the center of the fiber, completely covering its core (Figure 5c, see Methods and Supporting Information S14 for additional details). As a first step, we characterized the intensity of light coupled out of the flake fiber (regardless of its polarization) as a function of the injected linear polarization angle and compared it with the performance of an as-purchased bare fiber (Figure 5d, see Supporting Information S1 for the complete experimental setup). For the bare fiber, there is very little variation of the intensity with the azimuthal angle of injection (Figure 5e). Contrarily, the light coupled out of the flake fiber features a strongly polarized transmission, with a π periodicity we associate to the rotation of the injected polarization with respect to the MoOCl_2_ optical axes (Figure 5f). The finite value measured at the transmission minima arises from polarization dephasing within the fiber, which results in an uncontrolled ellipticity of light at the flake end of the fiber (see Supporting Information S15). Nevertheless, while the transmission through the bare fiber does not depend on the impinging polarization, the enhanced flake fiber shows a measured near-unity normalized polarization contrast in a broadband region from 500 to 1000 nm, thus demonstrating polarized transmission over the vis-NIR spectrum (Figure 5g). To thoroughly characterize the performance of the fabricated fiber, we analyze the polarization of the light coupled out of both the bare and flake fibers when the injected light is polarized along the [100] and [010] crystal axes of MoOCl_2_ (Figure 5h). The light transmitted through the bare fiber is always polarized, with maxima rotated by π/2 following the injected light (Figure 5i). The slight variation in maximum transmitted intensity can be attributed to marginal differences in injection efficiency into the fiber for different positions of the waveplate used to rotate the polarization (see Methods). Moreover, while the injected light is linearly polarized (see Supporting Information S15), the light passed through the fiber has elliptical polarization with a nonzero minimum intensity, thus confirming the aforementioned dephasing caused by the fiber. In contrast, the flake fiber shows linearly polarized transmission in case the injected light is polarized parallel to the [010] axis of the crystal (Figure 5j), with a ratio of maximum over minimum transmission larger than 2 × 10^2^. When the injected polarization is rotated of π/2, thus being parallel to the [100] crystal axis of the flake, the transmitted intensity has values ∼55 times smaller than what observed in the previous case. In addition, we tested the performance of the polarizing fiber when connected to a second optical fiber by a commercially available fiber connector, thus confirming the ready applicability of MoOCl_2_ as a miniaturized optical element (see Supporting Information S16). These observations confirm the successful fabrication of a broadband polarizing optical fiber by positioning a single MoOCl_2_ flake at the end of a standard nonpolarizing, nonpolarization maintaining SM optical fiber. Our solution provides a cost-effective and compact alternative to commercially available polarizing optical fibers, which involve more expensive fabrication processes, are often narrowband and rely on highly polarized absorption, thus requiring a minimum propagation length of light within the fiber to function effectively.

**Figure 5:**
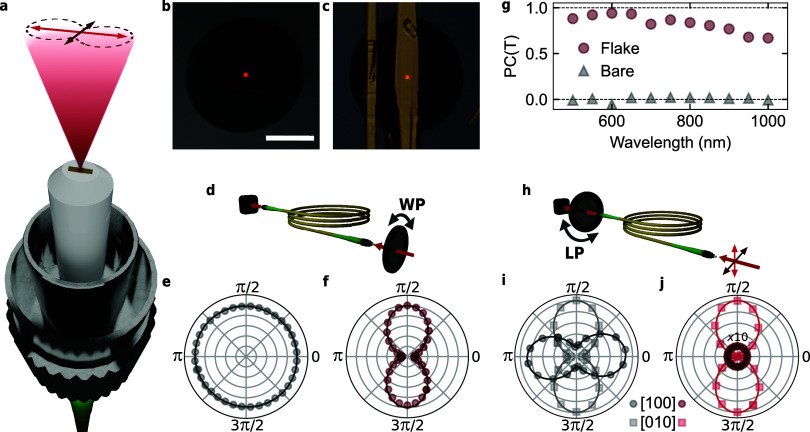
Realization of a broadband polarizing fiber through the integration of a MoOCl_2_ flake on its core. (a) Thin MoOCl_2_ flakes can be placed on fiber ends to create a broadband polarizing fiber. (b,c) Optical images of the fiber end without (b) and with (c) a MoOCl_2_ flake on it. Light at 650 nm wavelength is propagating in the core of the fibers. Scalebar is 50 μm. (d–f) When the input polarization is varied with a half waveplate before the fiber (WP, d), the intensity of light coupled out of a bare fiber is constant, regardless of the injected polarization (e). Conversely, the flake modulates the intensity of light coupled out of the fiber, polarizing it (f). (g) The fabricated polarizing fiber show large normalized PC (red dots), while the same fiber without a flake transmits the same intensity regardless of the impinging polarization (gray triangles). (h–j) When analyzed with a linear polarizer after the fiber (LP, h), light transmitted through the bare fiber largely retains the polarization of the input laser (i). In contrast, the flake fiber selectively transmits only one polarization (j). The two curves correspond to two perpendicular polarization of light injected in the fiber, parallel to the [100] (dots) and to the [010] (squares) crystal axes. The [100] transmitted intensity in (j) is multiplied by a factor 10 for clarity. Wavelength of light is 650 nm.

## Conclusions

In conclusion, we have characterized the in-plane optical tensor of MoOCl_2_, revealing an unprecedented degree of in-plane permittivity anisotropy |Re­(Δε_∥_)| > 10 at visible frequencies in a natural material. By introducing the parameter Δ_*P*_, we have demonstrated that in-plane hyperbolic crystals outperform conventional dielectrics in delivering high polarization contrast with minimal losses. We showed experimentally that values of Δ_*P*_ > 0.8 can be achieved in thin films of MoOCl_2_, pointing toward the realization of lossless polarization splitting. To further enhance the value of Δ_*P*_, improvements in both transmission and suppression of reflection along the dielectric axis are necessary. While enhancing transmission calls for materials with even lower optical losses, reducing reflection can be addressed by incorporating an antireflection coating, thereby relaxing the strict requirement of ε = 1. As exemplified by our study of the MoOCl_2_-based PBS and the demonstration of an ultrathin MoOCl_2_ polarizer integrated on an optical fiber, our findings underscore the potential of hyperbolic vdW crystals as promising candidates for the development of low-loss, miniaturized optical components for advanced polarization control in integrated photonic systems.

## Methods

### Mechanical Exfoliation and Dry Transfer

Commercially available wafers of fused SiO_2_ 500 μm thick from Wafer University were used as substrates. Before the exfoliation, the substrates were soaked in acetone for 5 min, then rinsed in isopropyl alcohol (IPA). To promote adhesion of the flakes, the substrates were cleaned in an oxygen-plasma cleaner for 15 min at maximum power. Flakes of MoOCl_2_ were exfoliated by the standard mechanical exfoliation technique from bulk crystals purchased from HQ Graphene. Commercially available PF Gel-Film ×4 from Gel-Pak was used. The exfoliated samples were left for 30 min in *N*-Methyl-2-Pyrrolidone to remove residues from the exfoliation, then bathed for 5 min in acetone and rinsed in IPA.

To transfer the flake onto the fiber end, a Nikon optical microscope was adapted as a manual transfer stage. Target flakes were picked up from the original exfoliation pad using a Gel-Film pad and then transferred onto the target fiber. All these procedures were done at room temperature and ambient conditions. Two samples were prepared: one using a P3–S405–FC fiber and the other using a P3–S630–FC fiber, both purchased from Thorlabs.

### Reflection and Transmission Measurements

A home-built optical microscopy system was used for the reflection and transmission measurements. A SuperK Extreme coupled to a SuperK Select filter from NKT Photonics were employed as a single-wavelength source tunable in two spectral windows from 400 to 650 nm and from 690 to 1100 nm. The incoming polarization was controlled by a linear polarizer and a broad-band half waveplate, both from Thorlabs. Polarized light was passed through a chopper (SR540 by Stanford Research Systems) and focused on the sample by a 20× Plan Apo objective (NA 0.42) from Mitutoyo. In reflection, light recollected from the same objective was focused on a silicon photodetector (PD, DET36A2 from Thorlabs) coupled to a SR830 lock in amplifier (LIA) from Stanford Research Systems, locked to the frequency of the chopper (383 Hz). In transmission, light was collected with a 50× Plan Apo objective (NA 0.55), also from Mitutoyo, and then focused on a second PD, connected to the same LIA. Imaging was performed in transmission with a white LED lamp (MWWHL4 from Thorlabs) and a RGB camera (The Imaging Source). For the measurements through the fiber, chopped light was injected in the free end of the fiber with a collimator package (PAF2-A7A from Thorlabs) and collected from the flake end of the fiber through a 10× Plan Apo objective from Nikon (NA 0.45). The light was then focused on the same PD used for transmission. The polarization was rotated before injection with a series of a polarizer and a half-waveplate, while the output of the fiber was analyzed with a polarizer before the PD. Optical imaging of the fibers (Figure 4b,d) was performed in the same setup. See Supporting Information S1 for the complete schematics of the two setups.

### Fitting of Reflection and Transmission Spectra

In Figure 2 we show fits of the reflection and transmission spectra along the [100] and [010] axes of a MoOCl_2_ crystal. From these fits we extract the experimental dielectric function. The optical response of the system is modeled by a three-layer system at normal incidence. The reflection *r* and transmission *t* Fresnel coefficients are calculated as^51^

2 $$r=\frac{r_{12}+r_{23}e^{2i\delta }}{1+r_{12}r_{23}e^{2i\delta }}$$

3 $$t=\frac{t_{12}t_{23}e^{i\delta }}{1+r_{12}r_{23}e^{2i\delta }}$$

where the *r*_*ij*_ and *t*_*ij*_ are Frensel coefficients of single interfaces

δ=2πdn/λrij=ni−njni+njtij=2njni+nj

here *d* and *n* are the MoOCl_2_ thickness and complex refractive index, respectively and λ is the wavelength. The indexes correspond to 1 for the material above the flake (air, *n* = 1), 2 for the MoOCl_2_ layer and 3 for the substrate (SiO_2_, *n* taken from ref 52). The intensity reflection and transmission are then calculated as *R* = |*r*|^2^ and *T* = |*t*|^2^/*n*_3_. We fit the dielectric function of both the MoOCl_2_ [100] and [010] axes with a Lorentz–Drude model

4 $$\varepsilon \left(\omega \right)=\varepsilon_{\infty }-\frac{\omega_{p}^{2}}{\omega \left(\omega +i\gamma \right)}+\sum_{j}\frac{\omega_{p,j}^{2}}{\omega_{0,j}^{2}-\omega^{2}-i\gamma_{j}\omega }$$

For the [100] direction we use a single Lorentzian contribution, while for the [010] one we use two Lorentzian but no Drude term (ω_p_ = 0). The parameters in eq 4 are the ones used to minimize the fit to the experimental data. For a robust estimation of the dielectric function parameters, we fit reflection and transmission spectra along a single axis at the same time with the same Drude-Lorentz values. We do this by stitching the reflection and transmission experimental data, which we fit with the concatenation of the calculated spectra from the three layer system. The thickness of the flakes is fixed by AFM measurements (see Supporting Information S4).

## Supplementary Material



## Notes

It contains additional information on the experimental setup, alignment procedures and characterization of the light beam polarization, simulations of the non-normal incidence optical response, AFM characterization, additional experimental curves, additional discussion on the dielectric function, plots of alternative metrics for anisotropic optical response, further comparison with other anisotropic materials, additional images, analysis and stress test of the optical fiber, thermal characterization of MoOCl_2_ (PDF)
