# Supplementary material for: Giant Optical Anisotropy in a Natural van der Waals Hyperbolic Crystal for Visible Light Low-Loss Polarization Control
Source: ACS Nano. 2025 Jul 1;19(27):25413–21. doi: 10.1021/acsnano.5c07323 (PMC12269362; doi:10.1021/acsnano.5c07323)
Supplement: Supplementary file 1 [file nn5c07323_si_001.pdf]

Giant optical anisotropy in a natural van der Waals hyperbolic crystal for visible light low-loss polarization control Supporting Information

Nicola Melchioni,[*,†,¶] Andrea Mancini,[*,†,¶] Lin Nan,[†] Anastasiia Efimova,[†,‡] Giacomo Venturi,[†] and Antonio Ambrosio[*,†]

†*Centre for Nano Science and Technology, Fondazione Istituto Italiano di Tecnologia, Via Rubattino 81, Milano 20134, Italy*

‡*Physics Department, Politecnico di Milano, Piazza Leonardo da Vinci 32, Milano 20134, Italy*

¶*These authors equally contributed to this work*

E-mail: nicola.melchioni@iit.it; andrea.mancini@iit.it; antonio.ambrosio@iit.it

S1 Experimental setup

The experimental setup we used consists of a home-built optical microscope. We employ SuperK Extreme coupled to SuperK Select from NKT Photonics as a tunable monochromatic source. Select has two emission windows, coupled to two different optical fibers: visible (vis), which can be tuned from 400 nm to 650 nm, and near-infrared (NIR), with a window from 690 nm to 1100 nm. Both light outputs are coupled in the system and expanded to 7 mm diameter beams. The two beams are joined in a single optical path using a short-pass dichroic mirror with a cutoff wavelength of 650 nm (DM1), then passed through a polarizer (P) and a chopper (CH). A 50:50 beam splitter (BS1) reflects light towards a broadband half waveplate (WP) and into the 20x objective (OBJ-R). In reflection, light recollected by OBJ-R passes again WP and BS1 and is focalized into the photodetector (PDR). In transmission, light is collected by a 50x objective (OBJ-T) and focalized into the photodetector (PDT).

Both the sample and the OBJ-T are mounted on a 3D micrometric stage to allow for navigation of the sample and precise alignment of the optical path. Imaging to navigate the sample is performed in transmission, using white light (LED) injected in OBJ-R, collected

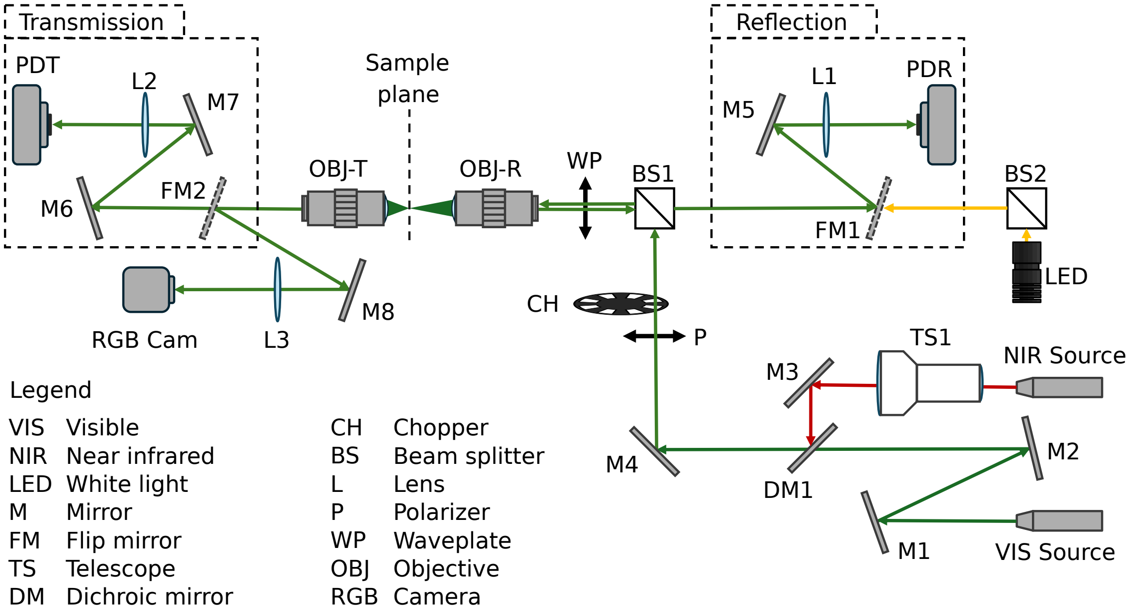

Figure S1: Schematics of the experimental setup.

by OBJ-T, and walked to a 3 color camera (RGB Cam) via a flip mirror (FM2) in the optical path. The sample plane is imaged on the camera through an achromatic lens (L3). The PDs are connected to an SR830 lock-in amplifier by Stanford Research Systems locked to the chopper frequency (383 Hz). The data are recorded by an automated script.

The system for measuring polarized transmission through the fibers is shown in Fig. S2. Light from the SuperK source is passed through a chopper, a polarizer (P1), and a waveplate (WP). The beam is then injected into the fiber through a commercial coupler. On the other side of the fiber, light coupled out of the ferrule is collected by a 10x objective which collimates it. For the linear dichroism measurements (Fig. 4c, e and f in the main text), light was directly focused on the PD. For the polarization analysis of the beam (Fig. 4g, h in the main text), a second polarizer (P2) was added in the beam path. Imaging of the ferrule end (Fig. 4b, d in the main text) was performed by adding a LED lamp and a beam splitter in the path, and replacing the PD with the RGB Cam.

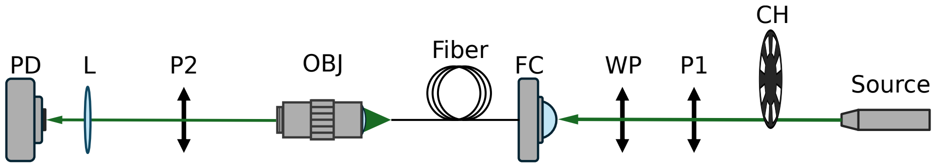

Figure S2: Schematics of the experimental setup for measurements through fiber.

S2 Procedure for aligning the polarization with the crystal axes

If the incident polarization is not properly aligned with the crystal axes, birefringence effects are introduced into the measurements, thus hindering an accurate determination of the dielectric tensor of the crystal. To rule out this effect from our experiments, we implemented a calibration procedure to minimize possible aliasing effects. The procedure consisted of three steps: first, the wavelength of excitation is fixed to a point where the anisotropy is large (630 nm for the vis range and 800 nm for the NIR range). Then, the transmission was measured as a function of the rotation angle of the waveplate, as shown in Fig. S3a. This results in a two-lobed curve (Fig. S3b) with a maximum for the angle corresponding to the dielectric axis, along which the transmission is high, and a minimum corresponding to the metallic axis, where the light is largely reflected (the equivalent in reflection, for which the two axes are exchanged, is reported in Fig. 2c in the Main text). Finally, we fitted the curve with the formula

$$I(\vartheta) = a + (c - a)\cos^2(\vartheta + b) \tag{S1}$$

where the parameter b corresponds to the angle at which the maximum signal is measured. We then measured the reflection and transmission across the whole spectrum by fixing the waveplate angle at $\vartheta_d = b$ for the dielectric axis, and at $\vartheta_m = b + \pi/4$ for the metallic one. We repeated such calibration for each different flake, and for each wavelength range.

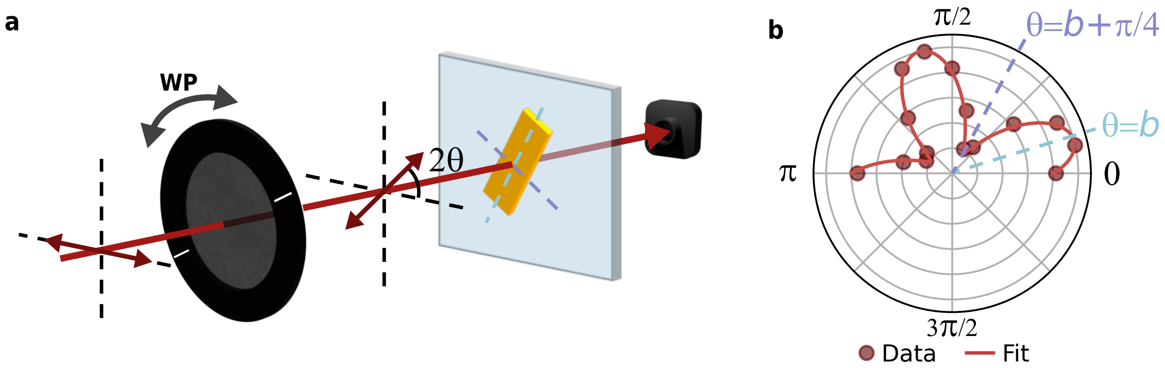

Figure S3: Procedure for the alinement with crystal axes. **a** At a fixed wavelength, rotating the waveplate sweeps the polarization impinging on the flake across the crystal axes. The transmission is then recorded as a function of the waveplate angle ϑ. **b** The transmission data (red dots) is fitted with Eq. S1 (red continuous line) to extrat the orientation angle b of the dielectric axis (maximum transmission, cyan dashed line). The orientation of the metal axis is consequently $b + \pi/4$ (purple dashed line).

S3 Optical response of $MoOCl_2$ at non-normal incidence

With the transfer matrix and the extracted dielectric function, the optical response at non-normal incidence can also be predicted (Fig. S4). As visible from the graphs, where we highlighted the maximum incidence angle of the light focused using the low-NA objective $\theta_R = \arcsin(0.42) \approx 25°$, we observe no drastic change in the reflection for both p and s polarized light along the [100] and [010] axes. The unvarying optical response at low-incidence angles justifies the fitting of the experimental data with a normal-incidence three-layer system model (see Methods in the main text).

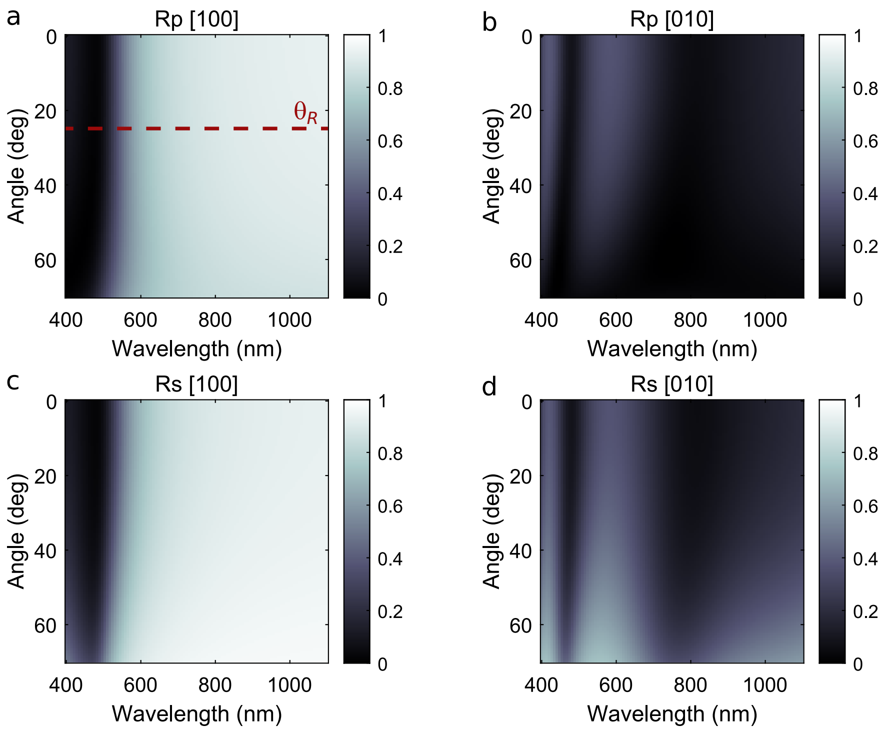

Figure S4: Calculated p-polarized **a, b** and s-polarized **c, d** reflectance along the [100] **a, c** and [010] **c, d** axes of a 173 nm $MoOCl_2$ flake. The dashed line corresponds to the maximum collection angle of the used low-NA objective $\theta_R \approx 25°$.

S4 Thickness and surface roughness determination via Atomic Force Microscopy

The Fresnel coefficients employed to compute reflection and transmission depend on the thickness of the $MoOCl_2$ layer (See Methods section in the main text). To reduce the number of free parameters in the fit for the dielectric function, we employed Atomic Force Microscopy (AFM) to retrieve the thickness of the analyzed flake. Fig. S5 show the AFM measurements taken on the flake used in the main text (Fig. S5a-c) and on a second, exemplary flake (Fig. S5d-f). The raw AFM data were analyzed with the software Gwyddion. The images were leveled by background subtraction, the thickness of the flake and the associated error are evaluated by using the Terraces tool in the software.

AFM can also be used to quantify the surface roughness of the measured flakes. Considering the data shown in Fig. S5, we extracted an average RMS roughness $\sigma_{rms} \sim 1\,\text{nm}$ on the flakes. Surface roughness can lead to partial scattering of the impinging power, with possible detrimental effects in the determination of the optical constants of the analyzed material. It is therefore important to evaluate the fraction of power lost due to surface roughness to eventually include it in the analytical model. Considering the extracted roughness value, in the spectral region of interest (400-1100 nm), it is always true that $\sigma_{rms} \ll \lambda$. Thus, the weak roughness limit holds, and it is possible to evaluate the fraction of power scattered by the surface roughness with the Bennett formula[1,2]

$$1 - \frac{R_{spec}}{R_0} = \left(\frac{4\pi\sigma_{rms}\cos(\vartheta_0)}{\lambda} \right)^2 \tag{S2}$$

where R_{spec} is the specular reflection, R_0 is the reflection of a perfectly smooth surface and ϑ_0 is the incident angle. According to the formula, for $\sigma_{rms} = 1\,\text{nm}$ and $\lambda = 400\,\text{nm}$ at normal incidence, the fraction of incident light is $< 10^{-3}$. Such a small value of power lost for scattering justifies the choice of neglecting surface roughness effects in the modelization

of the dielectric function.

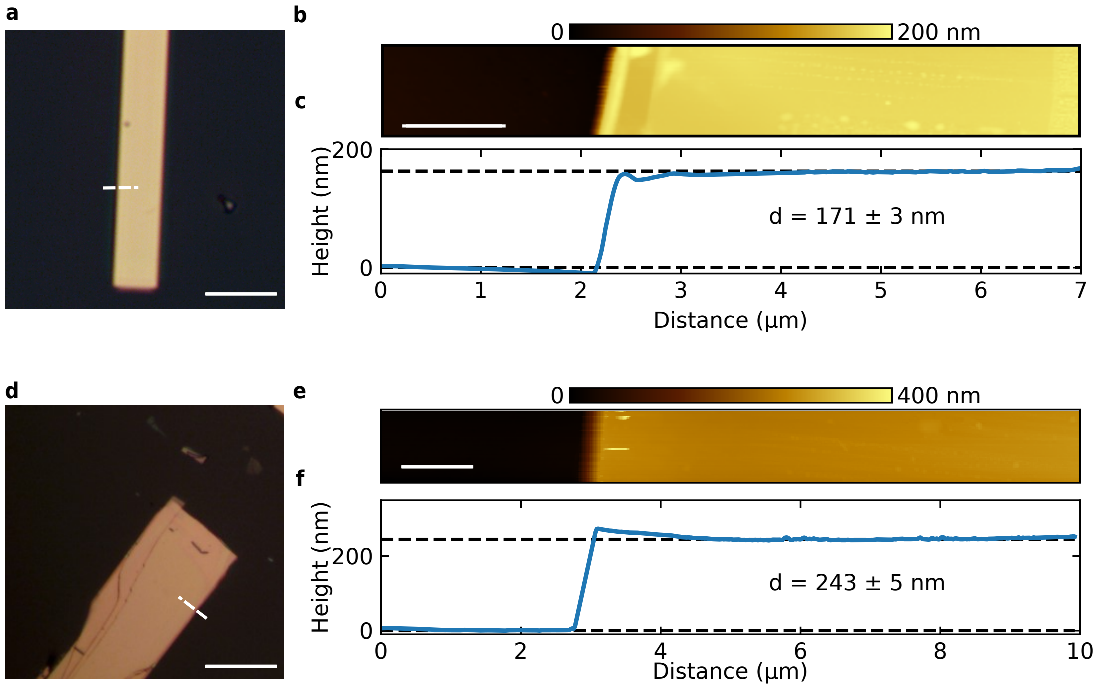

Figure S5: Atomic Force Microscopy measurements on two flakes employed in this work: the flake presented in the main text **a-c** and a second exemplary flake **d-f**. The scalebar in the optical images is 25 μm(**a**, **d**). The AFM scans are taken in the area highlighted with a white dashed line on the corresponding flake. The scalebar in the scans is 1 μm (**b**, **e**).

S5 Fits to additional flakes of various thicknesses and absorption

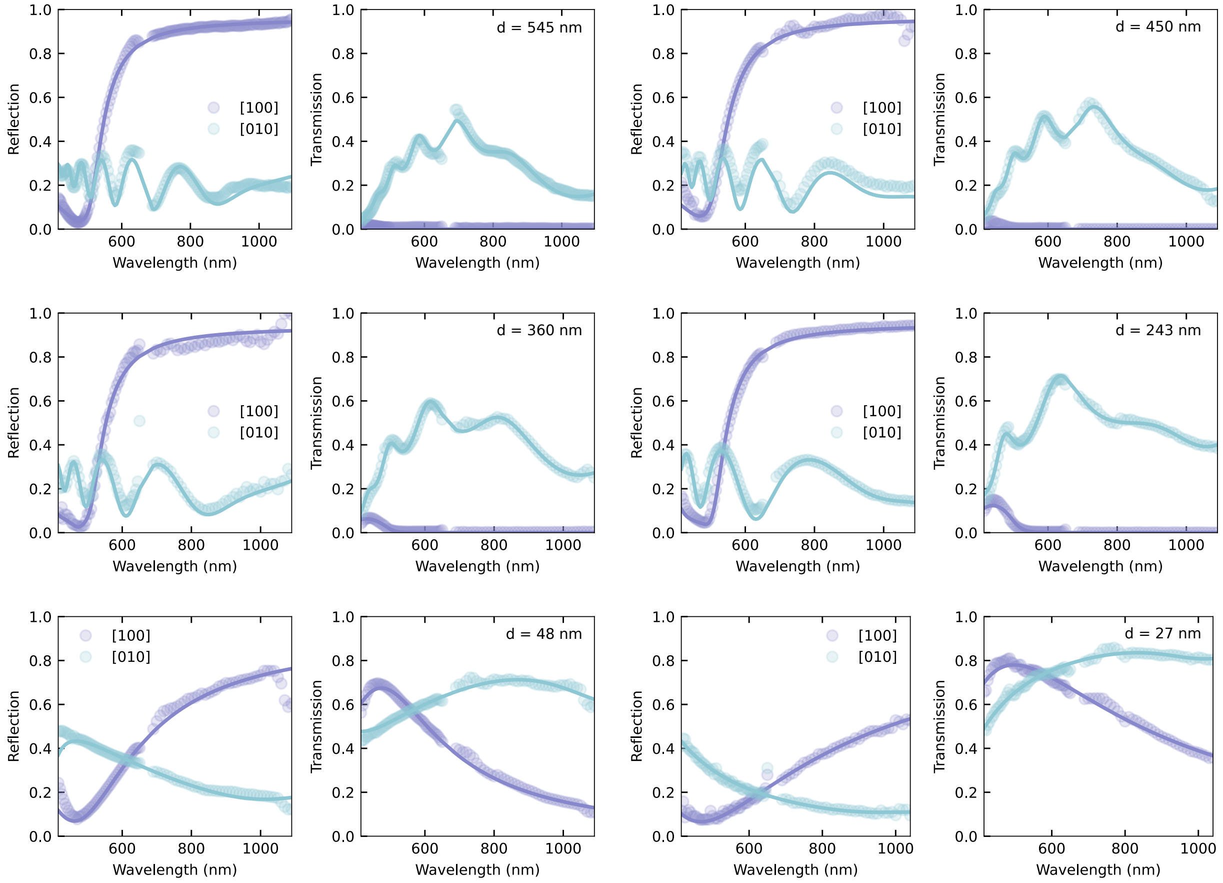

Figure S6: Additional example of fits to the reflection and transmission along the [100] and [010] axes for flakes of different thicknesses.

In Fig. S6 we report fits of the reflection and transmission of various $MoOCl_2$ flakes with different thicknesses. Having measured both the reflection and transmission of each flake, we can compute the absorption as $A = 1 - R - T$, which we show in Fig. S7 for selected flakes. Along the metallic [100] direction the absorption is low for longer wavelengths, and rises below 600 nm due to the loss of metallic behavior. In the dielectric side, losses are especially low in the middle part of the spectrum around 700 nm, but rise both above and below due to the presence of interband transitions.

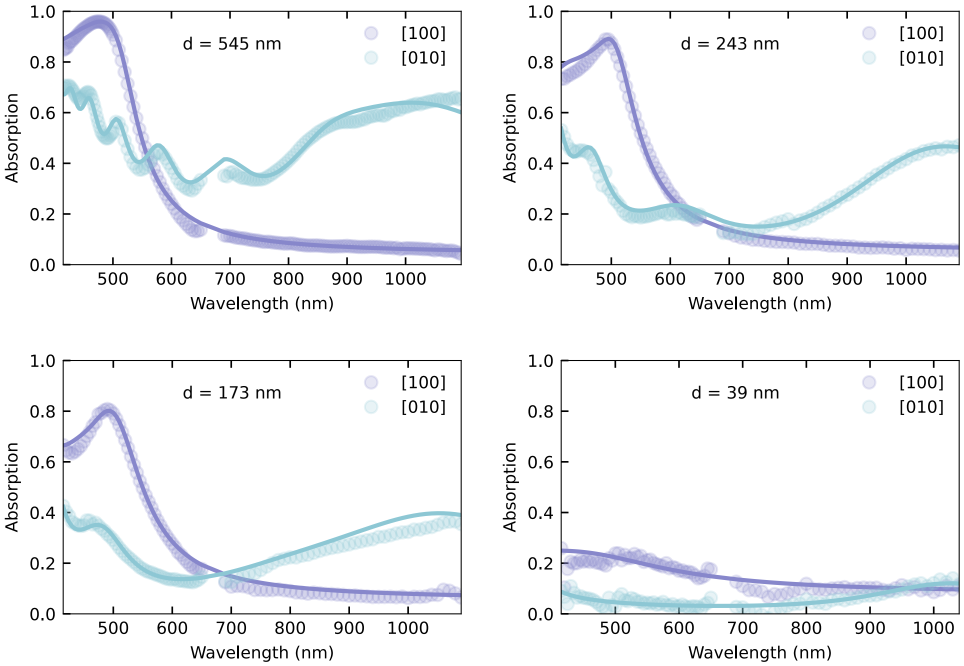

Figure S7: Absorption spectra for the [100] and [010] axes calculated as $A = 1 - R - T$ for flakes of different thicknesses. Continuous lines are computed from the fitted reflection and transmission curves. The flake with d = 173 nm is the one analyzed in the main text.

S6 Comparison of dielectric function from previous works

The dielectric function of $MoOCl_2$ has been reported before in literature.[3,4] In Ref.[3] it was calculated through density functional theory and is shown as dashed lines in Fig. S8. In Ref.[4] Ruta et. al, found that electron correlations strongly modify the band structure, leading to strongly damped interband transitions (dash-dotted lines in Fig. S8). In this latter case, the dielectric function was extracted from fit to the reflectivity of a thick flake. However, the measurement of only the reflectance of a single very thick flake over a very extended range prevented a precise extraction of the dielectric function. In our work we can better retrieve the optical response function as we use multiple flakes of various thicknesses and record at the same time both the reflection and transmission. In the following table, we report the values extracted from the fit.

Table S1: **Values of the parameters extracted from the fit**. All the values are reported in eV, except for ε_∞ which is a dimensionless parameter.

Axis	ε_∞	ω_p	γ	$\omega_{0,1}$	$\omega_{p,1}$	γ_1	$\omega_{0,2}$	$\omega_{p,2}$	γ_2
[100]	0	5.68	0.15	4.84	10.04	0.66	–	–	–
[010]	2.91	–	–	3.85	6.48	0.29	1.17	0.78	0.44

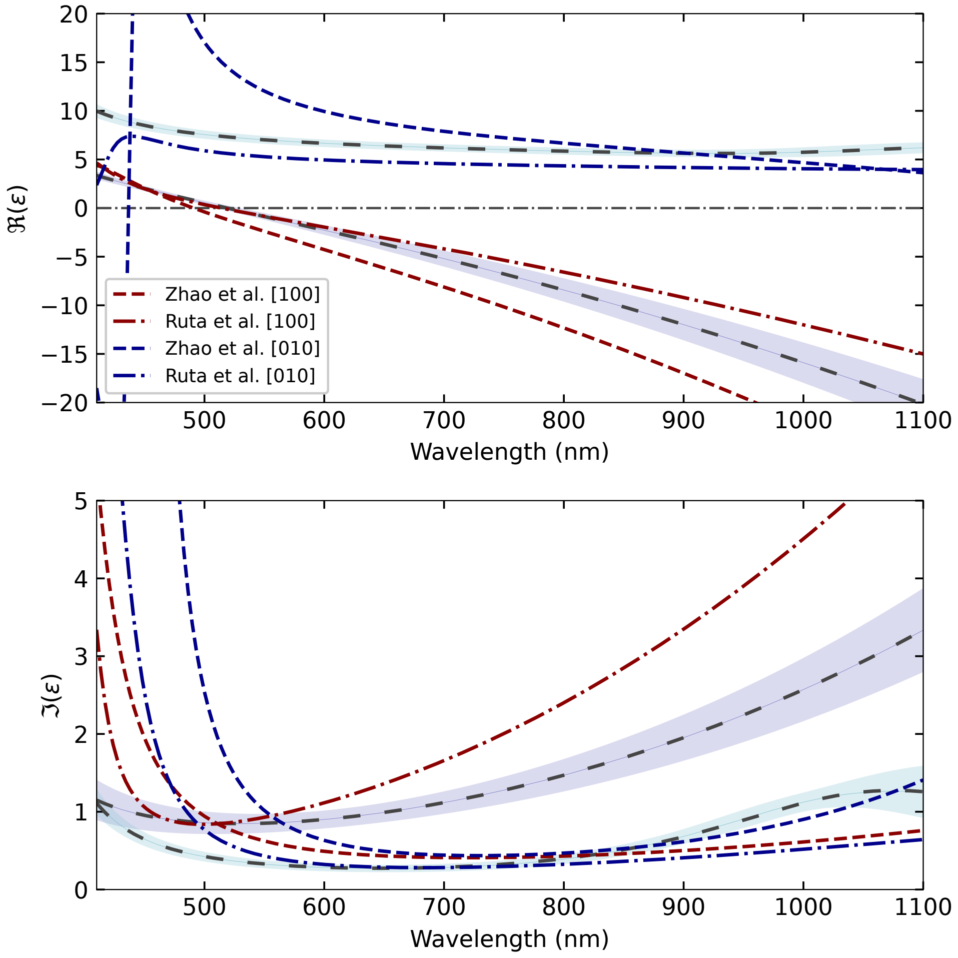

Figure S8: Comparison of the dielectric function extracted in our work with theoretical calculations from Ref.[3] and fit to experimental data in Ref.[4]

S7 Recalculation of reflection and transmission spectra from the extracted dielectric function

As a confirmation of the accuracy of the extracted dielectric function, we can compare the experimental data with the theoretical reflection and transmission spectra calculated with the new values of the dielectric function. The spectra are calculated as a three layer system, and the good agreement with the experimental data shown in Fig. S9 confirms the accuracy of the extracted dielectric function.

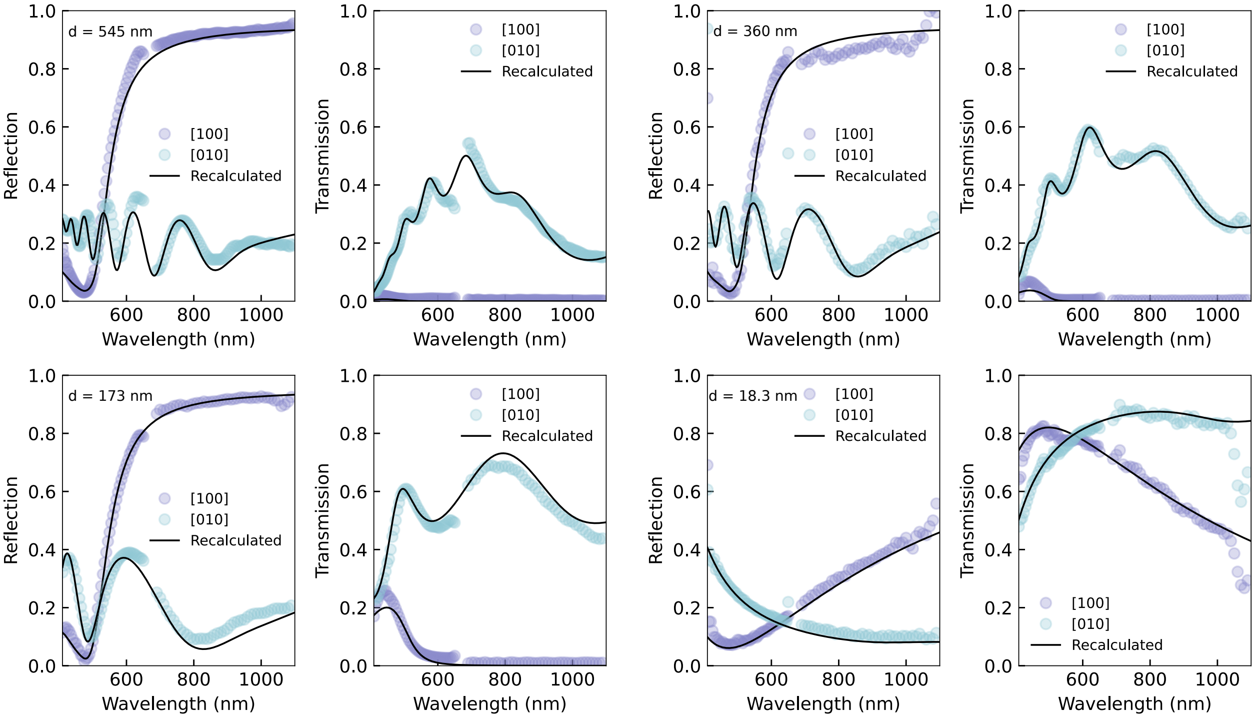

Figure S9: Examples of reflection and transmission spectra along the [100] and [010] axes recalculated from the extracted dielectric function obtained by averaging over all the fits.

S8 Comparison with near-field data on a 20 nm flake

As $MoOCl_2$ is a low-loss hyperbolic material it can host plasmon polaritons, which have been recently mapped in real space in thin flakes through scattering-scanning near optical microscopy (sSNOM).[4,5] As a further confirmation of the accuracy of the dielectric function extracted in this work, we can compare the predicted plasmon polariton dispersion with the experimental data reported on a 20 nm flake in Ref.[5] By overlaying the transfer matrix calculation with the sSNOM data, we observe very good agreement with the experimental dielectric function. Previously reported values of instead result in a worse agreement with the sSNOM experimental data.

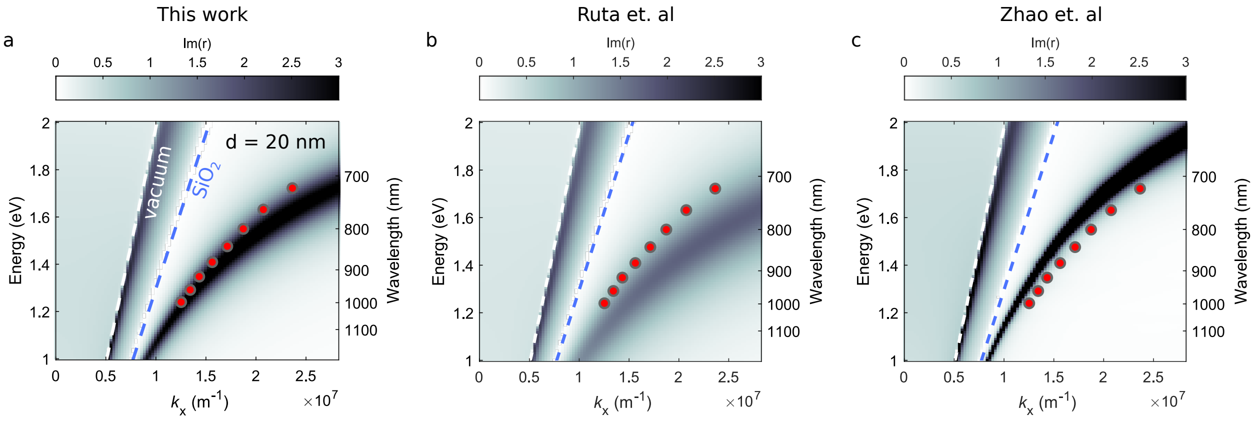

Figure S10: Comparison of the imaginary part of the Fresnel reflection coefficient calculated through the transfer matrix with the sSNOM data on a 20 nm flake reported in.[5] The calculations are carried out with the dielectric function extracted in this work **a**, the one used in Ref.[4] **b** and the one calculated in Ref.[3] **c**.

S9 Normalized polarization contrast in reflection and transmission

In Fig. 3 of the main text, we presented the anisotropic optical response of $MoOCl_2$, obtained from the measured reflection and transmission spectra. The extracted parameters effectively describe the differential reflection and transmission of the two polarizations, while also accounting for absorption within the material. However, in the literature, the normalized polarization contrast (or linear dichroism) in reflection is typically defined as $PC(R) = (R_x - R_y)/(R_x + R_y)$, with analogous definition for transmission. For a more complete comparison with other materials, in Fig. S11 we report the values of the normalized polarization contrast in reflection and transmission we extracted from the experimental data.

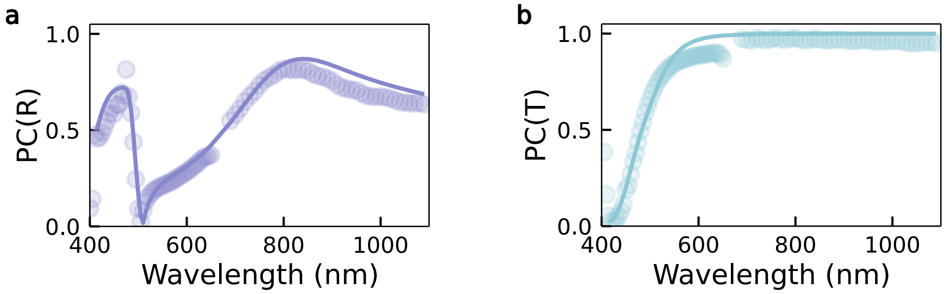

Figure S11: Experimental normalized polarization contrast in reflection (**a**) and in transmission (**b**) for a 173 nm thick flake of $MoOCl_2$.

The definition of normalized polarization contrast as the normalized difference in reflection or transmission along the two crystal axes relates to the degree of polarization of light after interacting with the material, but it neglects absorption within the material. In fact, it reaches a value of 1 as soon as the reflected or transmitted light along one axis drops to zero, even if only a minimal fraction of light is reflected or transmitted along the other axis. Indeed, the high normalized polarization contrast of many common birefringent materials are attributed to high absorption along one axis. For example, a 120 nm thick flake of $NbOCl_2$ - the highest reported dielectric birefringent material to date - presents $PC(T) = 0.99$ for an

illumination of 310 nm with and average absorption $< A >\sim 0.6$.[6] Conversely, the studied 173 nm $MoOCl_2$ flake reaches values of $PC(R) = 0.8$ and $PC(T) > 0.99$ with $< A >\sim 0.2$ in a broadband region from 600 nm to 1100 nm, showcasing the superior performance of hyperbolic materials over dielectric anisotropic ones.

S10 Plot of $|\Delta T|$ versus $|\Delta R|$

In the main text we propose Δ_P as a parameter that evaluates the optical anisotropy of materials while also taking into account the optical losses involved in the light-matter interaction. A value of $\Delta_P = 1$ clearly corresponds to the case where $R_x = T_y = 1$ with zero optical losses, but intermediate values cannot easily indicate wether the optical anisotropy is poor in reflection or in transmission. For a better visualization, a plot of $|\Delta R|$ versus $|\Delta T|$ can be used, as shown in Fig. S12. We included different anisotropic materials also studied in the main text. Each point on the curves is associated with a different wavelength, increasing from 400 nm to 1100 nm in steps of 65 nm, following the direction of the superimposed black arrows. In the plot, the origin corresponds to the isotropic case of $|\Delta R| = |\Delta T| = 0$, while a value of $\Delta_P = 1$ (bidirectional polarization contrast) corresponds to the top right angle, where $|\Delta R| = |\Delta T| = 1$. Anisotropic materials with values along the diagonal exhibit linear dichroism with zero losses, where anisotropy in reflection corresponds directly to the anisotropy in transmission, but outcoupled light is not perfectly polarized ($|\Delta R| = |\Delta T| \neq 0, 1$). In the rest of the quadrant, optical anisotropy in reflection and in transmission differ due to the effect of optical losses; however, in general, the closer the values are to the (1,1) corner, the lower the losses. By looking at Fig. S12, it is clear that $MoOCl_2$ and the other hyperbolic material $C_3H_8N_6I_6$ reach areas closer to (1,1) than the reported exemplary dielectric anisotropic materials that have either high losses ($NbOCl_2$) or modest optical anisotropy (TiO_2). Other materials considered in the main text were omitted for clarity of representation.

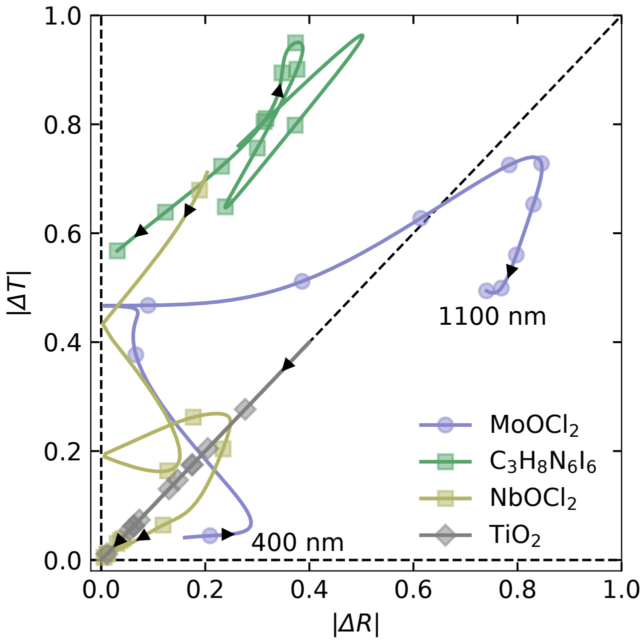

Figure S12: Plot of $|\Delta R|$ versus $|\Delta T|$ for three different materials reported in the literature.[6,7] The curves show values for 173 nm thick flakes at different wavelengths, from 400 nm to 1100 nm in steps of 65 nm. The wavelength increases linearly in the direction indicated by the superimposed black arrows.

S11 Material comparison through average normalized polarization contrast

In the main text we use $\Delta_P = (R_2 - R_1 - T_2 + T_1)/2$ as a measure of the optical anisotropy of a material. The advantage of this metric is that it is equal to 1 only in the case of no losses and complete reflection and transmission of orthogonal linear polarizations along distinct crystal axes. An alternative figure of merit can be the simple average of the polarization contrasts in reflection and transmission $< PC >= (PC(R) - PC(T))/2$. The comparison of $< PC >$ for several materials for a 173 nm film on glass (analogous to the one in Fig. 2 in the main text) shows that even with this different metric hyperbolic materials outperform standard dielectrics (Fig. S13). This is the case independently of the precise film thickness as shown in Fig. S14. It should be noted that $< PC >$ suffers from the same issue as the individual normalized polarization contrasts (or linear dichroism), i.e. the fact that complete suppression of reflection (transmission) along one crystal direction is enough to give $PC(R) = 1$ ($PC(T) = 1$), independently of the total amount of reflection (transmission). Therefore, while having $< PC >= 1$ guarantees a complete degree of polarization both in reflection and transmission, it does not necessarily implies no optical losses. As the absence of losses is important to evaluate the performance of a crystal for its use as an optical element, we prefer to report Δ_P rather than $< PC >$ in the main text.

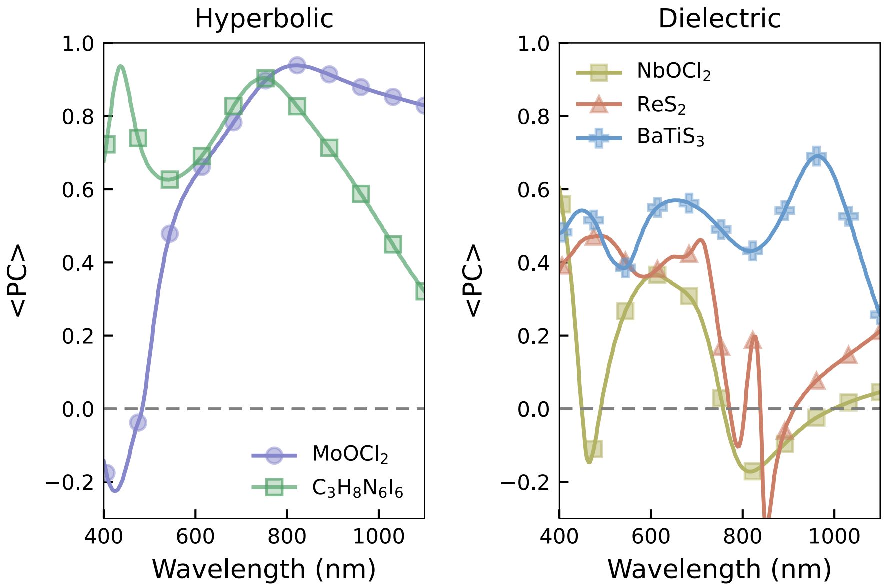

Figure S13: Comparison of $< PC >$ for in-plane birefringent materials for a 173 nm film on glass.

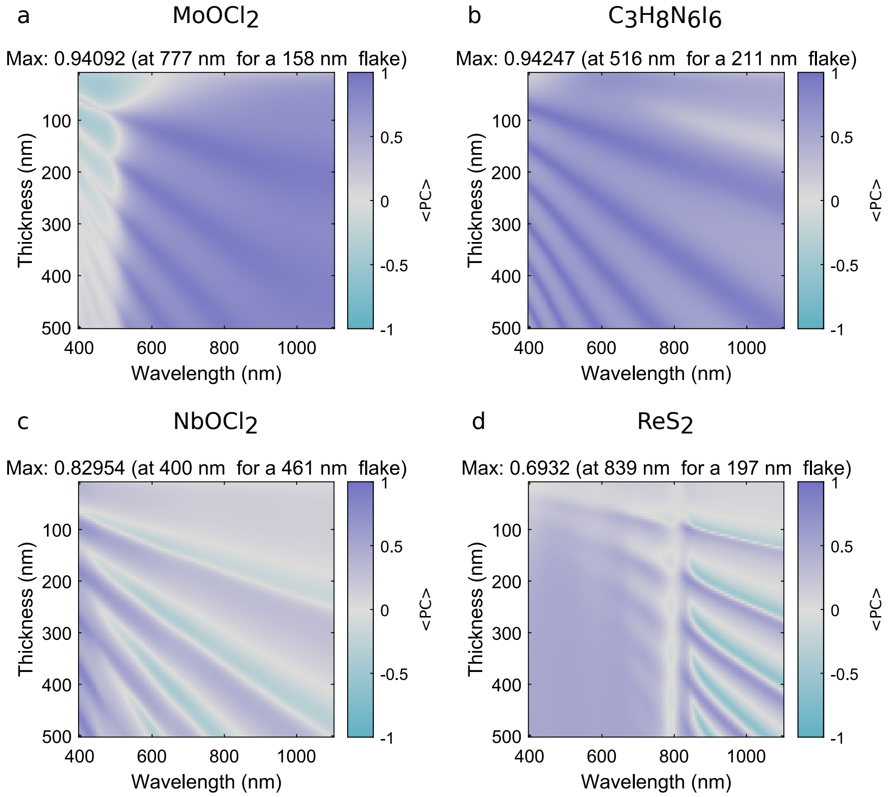

Figure S14: $< PC >$ at various film thicknesses for crystals showing highest in-plane birefringence across the visible and near-IR ranges. **a** $MoOCl_2$, **b** $C_3H_8N_6I_6$,[7] **c** $NbOCl_2$[6] and **d** ReS_2.[8]

S12 Optical response of in-plane anisotropic materials for various flake thicknesses

In Fig3 c-d of the main text we compare Δ_P for several materials, including both hyperbolic and dielectric ones for a 173 nm thickness. Here, we show how this metric varies as a function of the flake thickness. To evaluate Δ_P, both the reflection (i), (ii) and transmission (iii), (iv) along the two orthogonal crystal axes have to be computed, as shown for the hyperbolic materials $MoOCl_2$ and $C_3H_8N_6I_6$ [7] in Fig. S15. Additionally, we show the conventional normalized polarization contrast (both in reflection (iii) and transmission(vii)) and the simple difference between the optical response along the two orthogonal directions (both in reflection (iv) and transmission (viii)). Here x, y are respectively the $[100], [010]$ crystal axes in $MoOCl_2$.

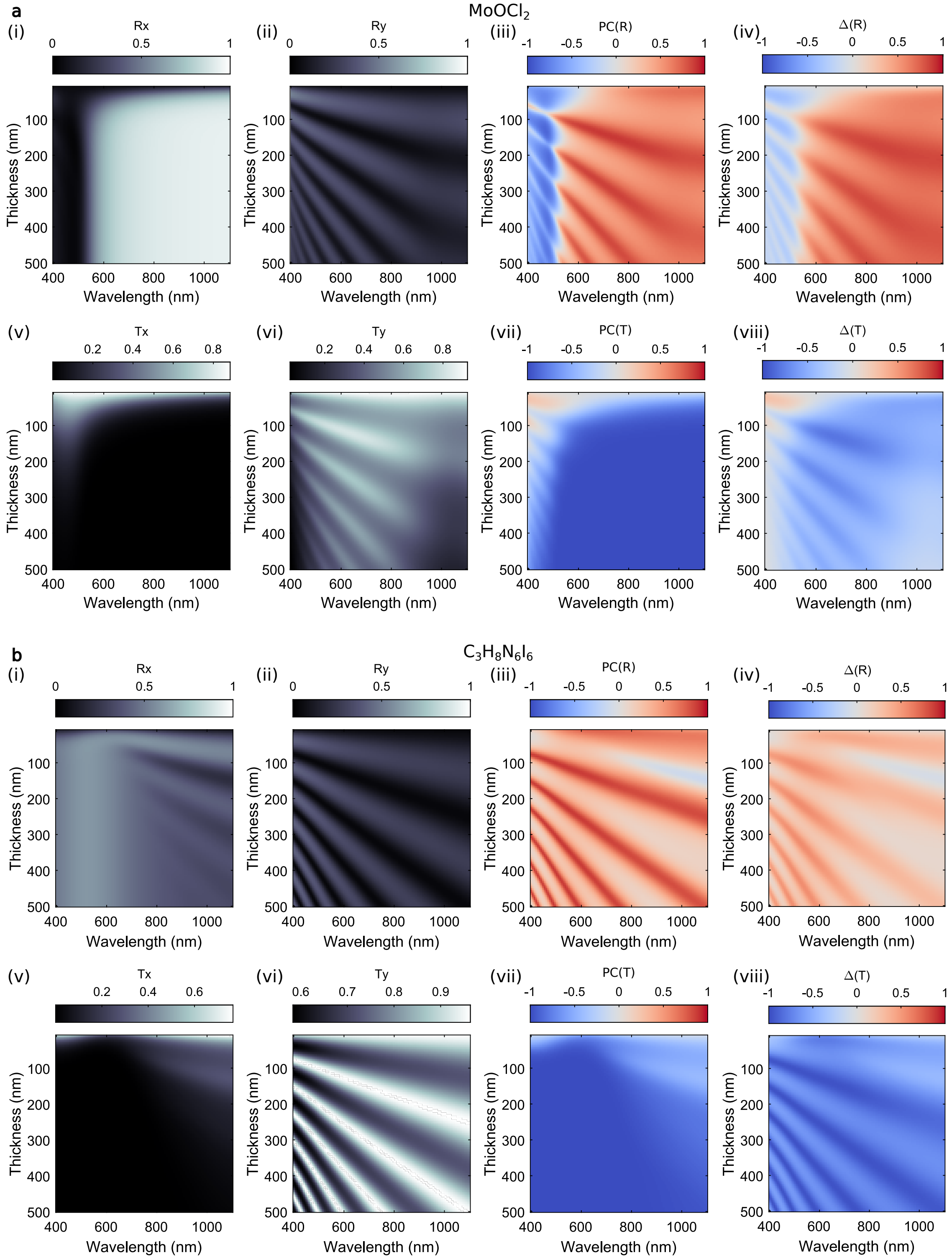

Figure S15: Transfer matrix simulations of the optical response of hyperbolic $MoOCl_2$ **a** and $C_3H_8N_6I_6$ **b**. For each material we calculate the reflectance (i), (ii) and transmission (v), (vi) along the metallic (i), (v) and dielectric (ii), (vi) axes of the crystal for various film thicknesses. With the calculated optical response we compute the reflectance PC(R) (iii) and transmission PC(T) (vii) normalized polarization contrast. We also show the corresponding in-plane differences of the reflectance (iv) and transmittance (viii).

From the calculated transmission and reflection, Δ_P can be calculated as a function of the flake thickness. Crystals with the highest in-plane birefringence are naturally the ones featuring the highest Δ_P (Fig. S16). The maximum value of Δ_P depends both on the flake thickness and the selected wavelength. The highest values are reached again by the hyperbolic crystals $MoOCl_2$ and $C_3H_8N_6I_6$. The two are complementary as they span different wavelength ranges, with $C_3H_8N_6I_6$ more suitable in the lower visible wavelength range, while $MoOCl_2$ is more efficient in the red part of the spectrum and at near-infrared frequencies.

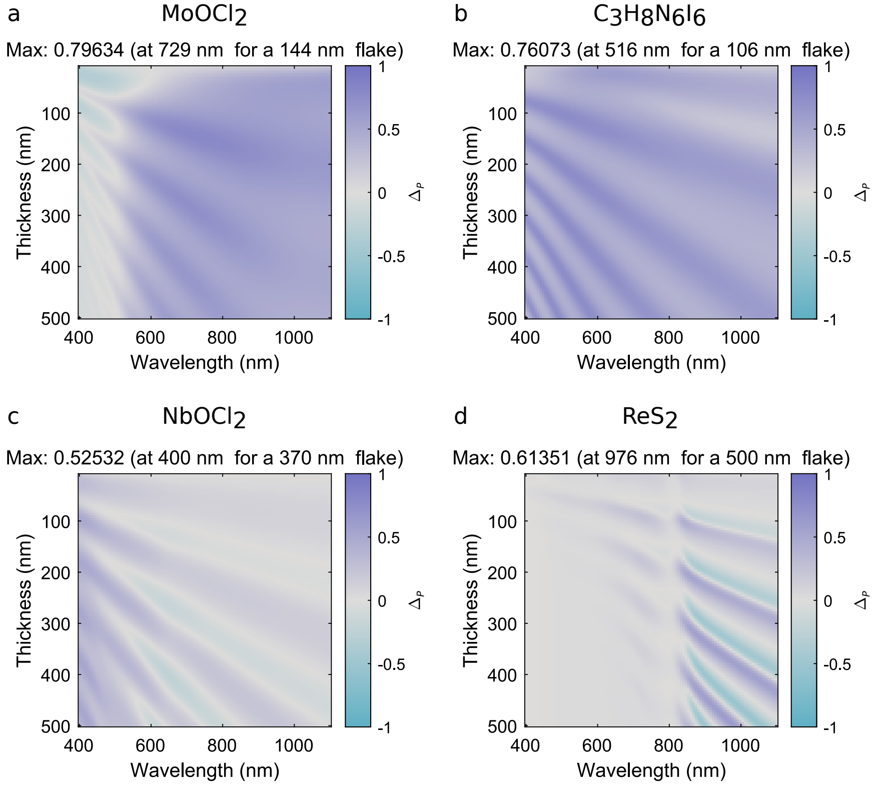

Figure S16: Δ_P at various film thicknesses for crystals showing highest in-plane birefringence across the visible and near-IR ranges. **a** $MoOCl_2$, **b** $C_3H_8N_6I_6$,[7] **c** $NbOCl_2$[6] and **d** ReS_2.[8]

S13 Birefringence comparison

In Fig. 3 of the main text we show the in-plane difference of the real part of the dielectric function as a way to evaluate the anisotropy of various materials. Although this can be useful to highlight the strength of hyperbolic materials, in the literature, birefringence (difference in the real part of the refractive index) and dichroism (difference in the imaginary part of the refractive index) are often reported. In Fig. S17 we show birefringence and dichroism for materials with the highest known in-plane anisotropy at visible and near-IR frequencies. $MoOCl_2$ has the highest reported values both in birefringence and dichroism, with the exception of $C_3H_8N_6I_6$ for the dichroism in the blue part of the spectrum. It should be noted that the high κ of $MoOCl_2$ in the near-IR region does not imply high losses along one crystal direction, as this occurs for the metallic axis, where the high κ does not result in absorption, as most light is reflected.

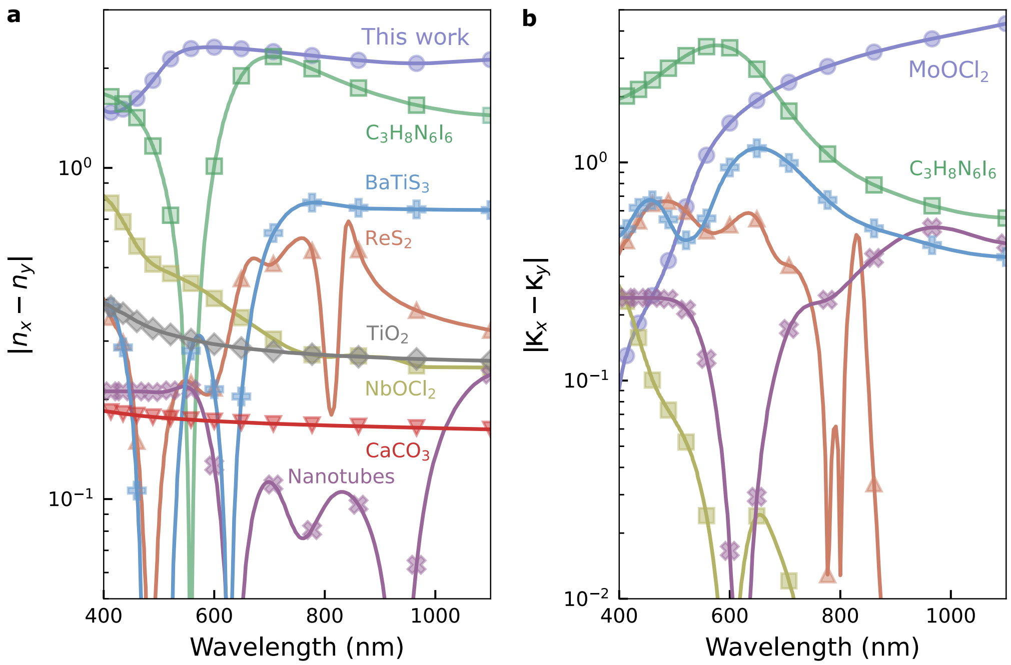

Figure S17: Birefringence **a** and dichroism **b** for several in-plane highly anisotropic crystals. Values of the refractive index are taken from the literature.[6–12]

S14 Additional images of the fibers end

In Fig. 4 of the main text we showed the end of a bare fiber and the flake fiber we fabricated. To support our claim of broadband transmission, here we report the same images for different illumination conditions. Fig. S18a-d show the transmission of the bare fiber we used. Fig. S18e is taken with laser off. S18f-i show that light polarized parallel to the [010] axis of the flake we deposited is transmitted for all wavelengths spanning from 480 nm to 630 nm. When the polarization is rotated parallel to the [100] axis, no light is transmitted through the flake (Fig. S18j).

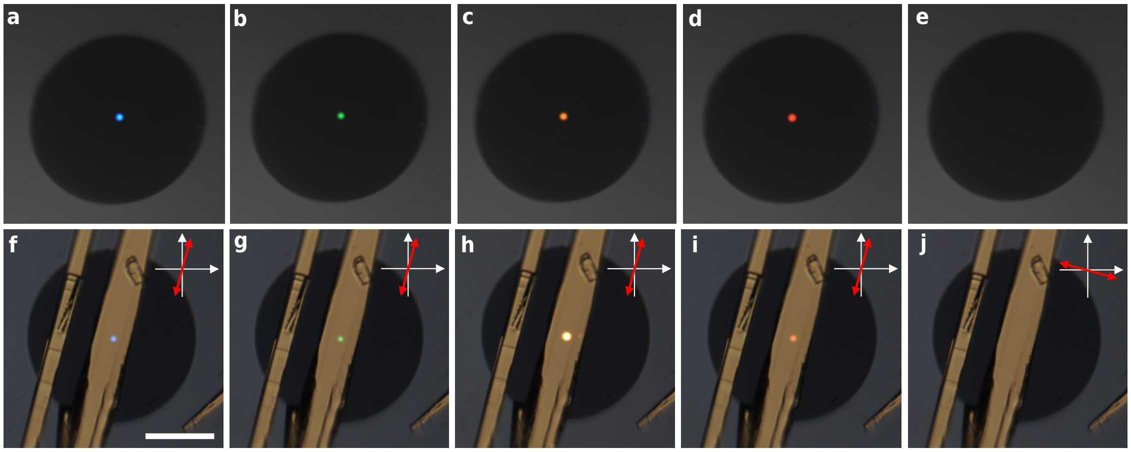

Figure S18: Optical images of the fiber end. **a-e**, Images of the bare optical fiber end for different condition of illumination: 480 nm (**a**), 550 nm (**b**), 600 nm (**c**), 630 nm (**d**) and light off (**e**). **f-j**, Images of the flake optical fiber end for different condition of illumination, as above. Polarization of the injected light is sketched in the inset: when the polarization is parallel to the [010] axis of $MoOCl_2$ light is transmitted. Contrarily, no light is passed when polarization is rotated parallel to [100] axis (**j**).

S15 Polarization of the beam in free space and in the fiber

All the conclusions we drawn so far are based on the assumption that the impinging light is fully polarized. To ensure this, we measured the polarization ratio of the free propagating beam after the first polarizer. We analyze light passing through a polarizer (P1 in Fig. S19a) by rotating its polarization with a half wave plate (WP), and we measure the transmission through a second polarizer (P2). The input beam is strongly polarized (Fig. S19b). The angular dependence of the light passed through the polarizer is fitted with Eq. S1. The polarization extinction ratio (ER), defined as the ratio of intensity along the transparent axis to that along the perpendicular axis $ER = I_{max}/I_{min}$, can be determined using the ratio c/a of the fitted parameters. Using this approach, we measure an $ER \sim 250$, compatible with the manufacturer specifications for P1.

To complete the characterization, we also analyzed the light coming out of the bare fiber for various illumination wavelengths (Fig. S20a). We fitted all the measured curves with Eq. S1 to extract the polarization parameters. Since the fiber is a standard step-index, the polarization of the propagating light is strongly affected by the specific strain and curvature of the fiber and also by the wavelength. As a result, ER varies with the wavelength without a specific trend (Fig. S20b), while the maximum polarization is rotated following a linear trend (Fig. S20c) that reflects the variations of the index of refraction with the wavelength.

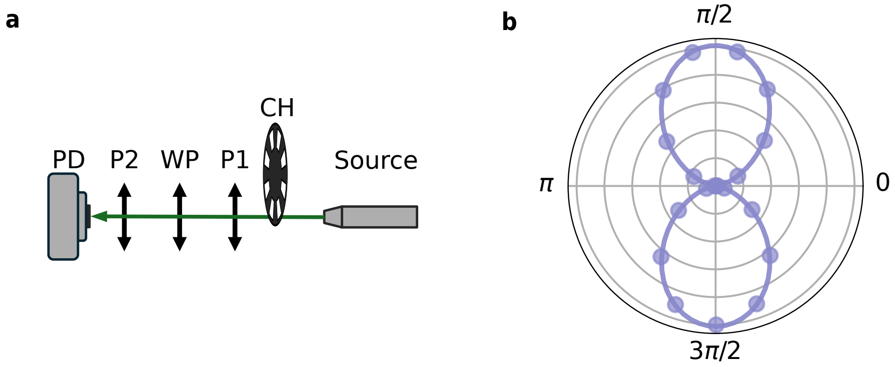

Figure S19: Polarization properties of the input beam. **a** Schematics of the setup used for the measurement. **b** Intensity transmitted through P2 as a function of the azimuthal angle of WP in **a**. The dots are measured points, while the continuous line is a fit with Eq. S1

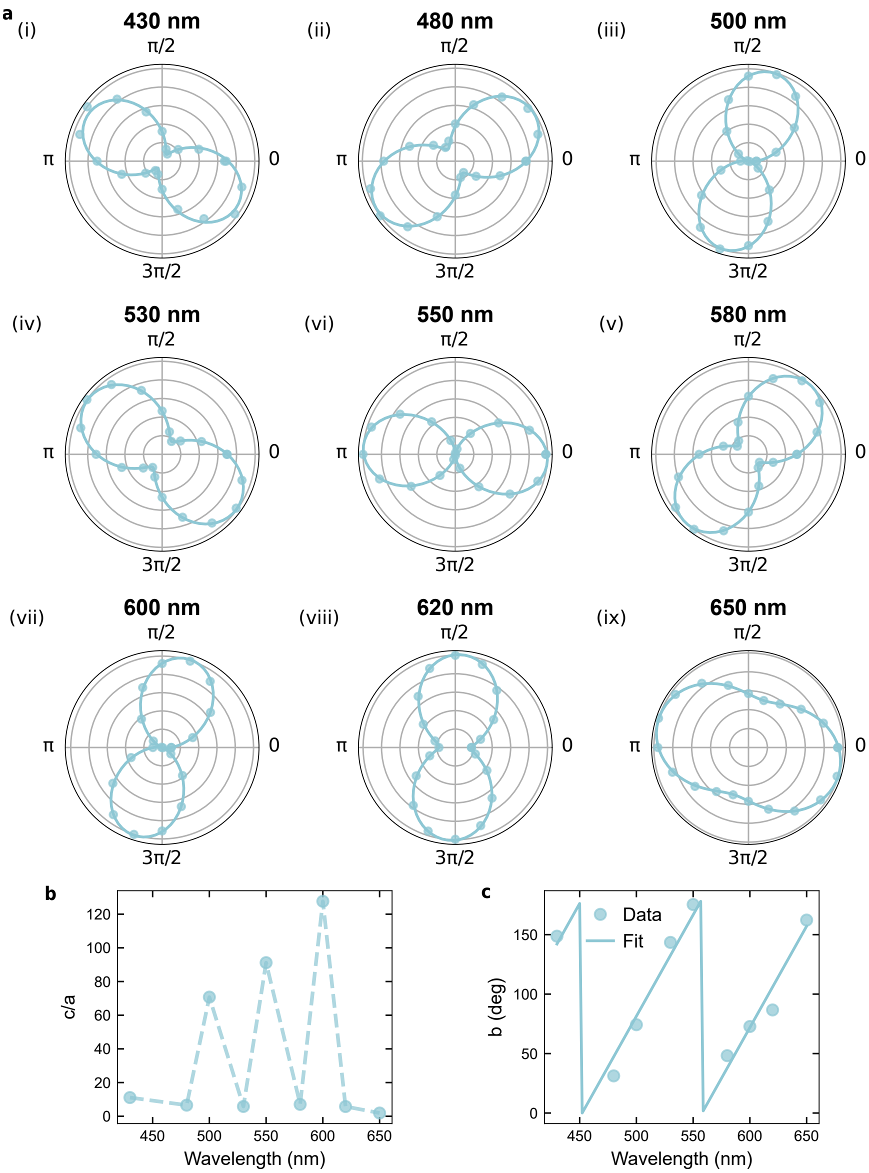

Figure S20: **a**, Polarization of light transmitted through a fiber for different illumination wavelengths and a fixed input polarization. The continuous line is a fitting with Eq. S1. **b**, Polarization extinction ratio as computed from the fit. The dashed line is a guide to the eye. **c**, Rotation of the polarization angle as a function of the illumination wavelength. The input polarization is fixed along the horizontal axis. The continuous line is a linear fit.

S16 Transmission through a second fiber connected to the flake fiber

To prove the unique and stable integrability that $MoOCl_2$ can provide, we connected the flake fiber presented in Fig. 4 of the main text to a second fiber without $MoOCl_2$ through a commercially available APC/APC fiber connector (ADAFC3 from Thorlabs, Fig. S21a). These connectors are designed for compactness while minimizing losses during light transmission between fibers, with an insertion loss of less than 0.5 dB according to the manufacturer's specifications. To achieve this, the connector features a support structure that aligns the ferrules of the two fibers and, according to the manufacturer description, ensures physical contact between them to minimize back-reflection, as illustrated in Fig. S21b. This condition makes it impossible to use standard optical components, as their use would require collimating the beam out of the fiber, passing light through the optics, and then coupling it back into the second fiber. In contrast, thin $MoOCl_2$ flakes placed on the fiber ferrule cause minimal physical alterations while significantly modifying the optical response, thereby enabling connection with other fibers and integration with existing components. To test this, we measured the transmission through the two connected fibers in the same setup sketched in Fig. S2. As shown in Fig. S21c, transmission remains strongly polarized even through the second fiber, exhibiting high DoLP values across a broad region of the vis-NIR spectrum (Fig. S21d). This confirms the successful integration of the two fibers into a single broadband polarizing fiber. The polarization direction shifts and the polarization ratio diminishes due to dephasing effects in the second fiber (see Supporting Information S15). These effects can be easily mitigated by using a polarization-maintaining fiber after the connection instead of a standard one. Our measurement demonstrates that $MoOCl_2$ can be employed to fabricate durable and versatile ultra-thin optical components for manipulating the polarization of light, offering a cheaper and superior alternative to the standard options available on the market.

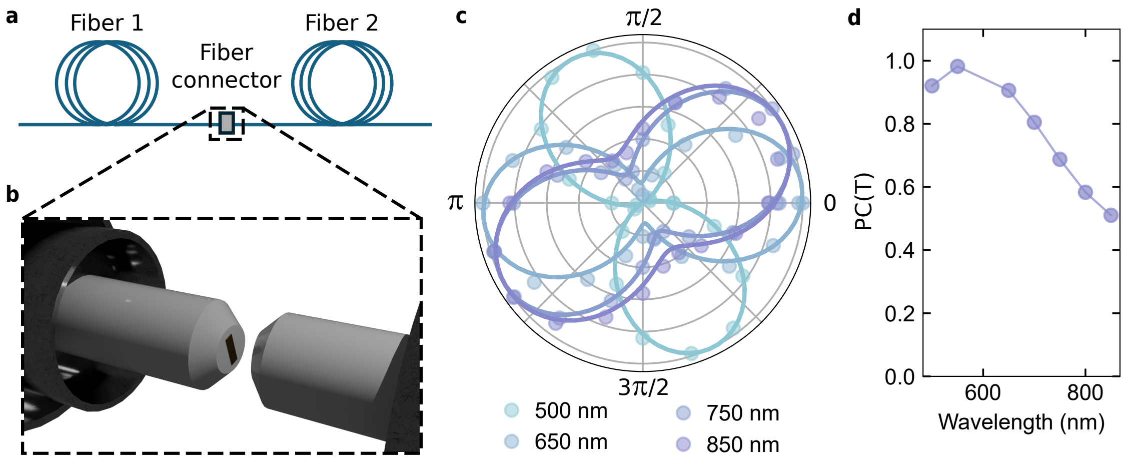

Figure S21: Connecting the polarized fiber to a second fiber. **a**, Schematics of the connection of two fibers through a commercially available APC/APC connector. **b**, The connector aligns the ferrules of fiber 1 (flake fiber) and fiber 2 close to each other. **c**, Transmission of light through the second fiber as a function of the polarization angle of the injected light for different light wavelengths. **d**, Normalized PC of the light passing through the fiber-flake-fiber system.

S17 Thermal stability of $MoOCl_2$

In addition to mechanical stability, discussed in the previous section, thermal stability is also an important parameter. Although all experiments presented in this work were conducted at room temperature, it is important to evaluate the thermal stability of $MoOCl_2$ to rule out potential degradation during device fabrication or operation. To this end, we placed a commercial hot plate below a custom-built vertical microscope equipped with a polarizer and a RGB camera for imaging. With this setup, we inspected $MoOCl_2$ samples exfoliated on SiO_2 at various temperatures, ranging from room temperature to 300 °C in steps of 50 °C. Before taking each image, we allowed the crystal to thermalize for 5 minutes at the selected temperature. As shown in Fig. S22, the crystal remains stable up to approximately 300 °C. However, when held at 300 °C for several minutes, the flakes begin to change, becoming more transparent and exhibiting altered optical properties. In particular, the material becomes optically isotropic and its hyperbolic response is lost. However, given the relatively high temperature at which this modification occurs, we can reasonably conclude that $MoOCl_2$ is thermally stable for room temperature applications. In addition to our experiments, the previous literature showed applications of $MoOCl_2$ in devices fabricated with lithography processes[5,13] that involved hard baking at temperatures around 160 °C. Even in those cases, the fabrication did not hinder the optical properties of MoOCl2, thus confirming the stability of the presented material in commonly employed fabrication processes.

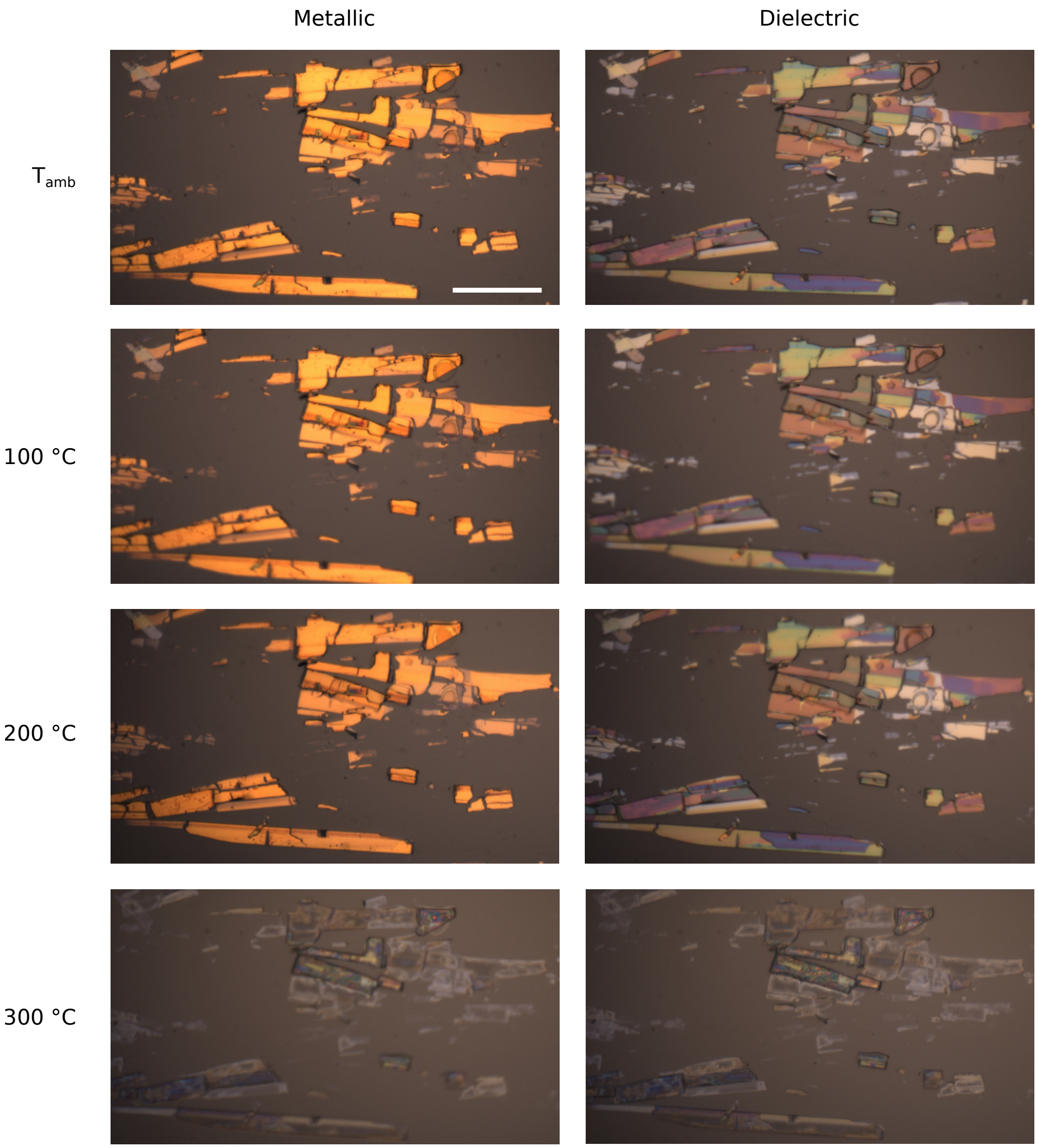

Figure S22: Temperature stability of $MoOCl_2$ in air. The material is thermally stable until around 300 °C. When left few minutes at 300 °C, it degrades and becomes isotropic. Images in the left (right) column are taken with polarization along the metallic (dielectric) axis. The scalebar is 50 µm

References

1. Bennett, H. E.; Porteus, J. Relation between surface roughness and specular reflectance at normal incidence. *Journal of the optical society of America* **1961**, *51*, 123–129.

2. Maradudin, A. A. *Light scattering and nanoscale surface roughness*; Springer Science & Business Media, 2010.

3. Zhao, J.; Wu, W.; Zhu, J.; Lu, Y.; Xiang, B.; Yang, S. A. Highly anisotropic two-dimensional metal in monolayer MoOCl 2. *Physical Review B* **2020**, *102*, 245419.

4. Ruta, F. L.; Shao, Y.; Acharya, S.; Mu, A.; Jo, N. H.; Ryu, S. H.; Balatsky, D.; Su, Y.; Pashov, D.; Kim, B. S.; others Good plasmons in a bad metal. *Science* **2025**, *387*, 786–791.

5. Venturi, G.; Mancini, A.; Melchioni, N.; Chiodini, S.; Ambrosio, A. Visible-frequency hyperbolic plasmon polaritons in a natural van der Waals crystal. *Nature Communications* **2024**, *15*, 9727.

6. Guo, Q.; Zhang, Q.; Zhang, T.; Zhou, J.; Xiao, S.; Wang, S.; Feng, Y. P.; Qiu, C.-W. Colossal in-plane optical anisotropy in a two-dimensional van der Waals crystal. *Nature Photonics* **2024**, 1–6.

7. Zhou, Y.; Guo, Z.; Gu, H.; Li, Y.; Song, Y.; Liu, S.; Hong, M.; Zhao, S.; Luo, J. A solution-processable natural crystal with giant optical anisotropy for efficient manipulation of light polarization. *Nature Photonics* **2024**, 1–6.

8. Munkhbat, B.; Wróbel, P.; Antosiewicz, T. J.; Shegai, T. O. Optical constants of several multilayer transition metal dichalcogenides measured by spectroscopic ellipsometry in the 300–1700 nm range: high index, anisotropy, and hyperbolicity. *ACS photonics* **2022**, *9*, 2398–2407.

9. Lynch, J.; Smith, E.; Alfieri, A.; Song, B.; Klein, M.; Stevens, C. E.; Chen, C. Y.; Lawrence, C. F.; Kagan, C. R.; Gu, H.; others Gate-tunable optical anisotropy in wafer-scale, aligned carbon nanotube films. *Nature Photonics* **2024**, *18*, 1176–1184.

10. Niu, S.; Joe, G.; Zhao, H.; Zhou, Y.; Orvis, T.; Huyan, H.; Salman, J.; Mahalingam, K.; Urwin, B.; Wu, J.; others Giant optical anisotropy in a quasi-one-dimensional crystal. *Nature Photonics* **2018**, *12*, 392–396.

11. Ghosh, G. Dispersion-equation coefficients for the refractive index and birefringence of calcite and quartz crystals. *Optics communications* **1999**, *163*, 95–102.

12. DeVore, J. R. Refractive indices of rutile and sphalerite. *JOSA* **1951**, *41*, 416–419.

13. Wang, Z.; Huang, M.; Zhao, J.; Chen, C.; Huang, H.; Wang, X.; Liu, P.; Wang, J.; Xiang, J.; Feng, C.; others Fermi liquid behavior and colossal magnetoresistance in layered MoOCl 2. *Physical Review Materials* **2020**, *4*, 041001.
